# Stem cell architecture drives myelodysplastic syndrome progression and predicts response to venetoclax-based therapy

**DOI:** 10.1038/s41591-022-01696-4

**Published:** 2022-03-03

**Authors:** Irene Ganan-Gomez, Hui Yang, Feiyang Ma, Guillermo Montalban-Bravo, Natthakan Thongon, Valentina Marchica, Guillaume Richard-Carpentier, Kelly Chien, Ganiraju Manyam, Feng Wang, Ana Alfonso, Shuaitong Chen, Caleb Class, Rashmi Kanagal-Shamanna, Justin P. Ingram, Yamini Ogoti, Ashley Rose, Sanam Loghavi, Pamela Lockyer, Benedetta Cambo, Muharrem Muftuoglu, Sarah Schneider, Vera Adema, Michael McLellan, John Garza, Matteo Marchesini, Nicola Giuliani, Matteo Pellegrini, Jing Wang, Jason Walker, Ziyi Li, Koichi Takahashi, Joel D. Leverson, Carlos Bueso-Ramos, Michael Andreeff, Karen Clise-Dwyer, Guillermo Garcia-Manero, Simona Colla

**Affiliations:** 1 Department of Leukemia, The University of Texas MD Anderson Cancer Center, Houston, TX USA; 2 Molecular Biology Institute, University of California, Los Angeles, Los Angeles, CA USA; 3 Division of Rheumatology, Department of Internal Medicine, Michigan Medicine, University of Michigan, Ann Arbor, MI USA; 4 Department of Medicine and Surgery, University of Parma, Parma, Italy; 5 Department of Bioinformatics and Computational Biology, The University of Texas MD Anderson Cancer Center, Houston, TX USA; 6 Department of Genomic Medicine, The University of Texas MD Anderson Cancer Center, Houston, TX USA; 7 Department of Biostatistics, The University of Texas MD Anderson Cancer Center, Houston, TX USA; 8 Department of Hematopathology, The University of Texas MD Anderson Cancer Center, Houston, TX USA; 9 AbbVie Oncology Discovery, Chicago, IL USA; 10 Department of Stem Cell Transplantation and Cellular Therapy, The University of Texas MD Anderson Cancer Center, Houston, TX USA; 11 McDonnell Genome Institute, Washington University in St. Louis, St. Louis, MO USA; 12 Istituto Romagnolo per lo Studio dei Tumori ‘Dino Amadori’, Meldola, Italy

**Keywords:** Stem cells, Cancer stem cells

## Abstract

Myelodysplastic syndromes (MDS) are heterogeneous neoplastic disorders of hematopoietic stem cells (HSCs). The current standard of care for patients with MDS is hypomethylating agent (HMA)-based therapy; however, almost 50% of MDS patients fail HMA therapy and progress to acute myeloid leukemia, facing a dismal prognosis due to lack of approved second-line treatment options. As cancer stem cells are the seeds of disease progression, we investigated the biological properties of the MDS HSCs that drive disease evolution, seeking to uncover vulnerabilities that could be therapeutically exploited. Through integrative molecular profiling of HSCs and progenitor cells in large patient cohorts, we found that MDS HSCs in two distinct differentiation states are maintained throughout the clinical course of the disease, and expand at progression, depending on recurrent activation of the anti-apoptotic regulator BCL-2 or nuclear factor-kappa B-mediated survival pathways. Pharmacologically inhibiting these pathways depleted MDS HSCs and reduced tumor burden in experimental systems. Further, patients with MDS who progressed after failure to frontline HMA therapy and whose HSCs upregulated BCL-2 achieved improved clinical responses to venetoclax-based therapy in the clinical setting. Overall, our study uncovers that HSC architectures in MDS are potential predictive biomarkers to guide second-line treatments after HMA failure. These findings warrant further investigation of HSC-specific survival pathways to identify new therapeutic targets of clinical potential in MDS.

## Abstract (web summary)

Extensive characterization of the stem and progenitor cell hierarchies of myelodysplastic syndromes reveals compensatory survival mechanisms underpinning the failure of hypomethylating agents, and uncovers biomarkers that predict second-line clinical response to venetoclax-based therapy.

## Main

MDS arise from a small population of disease-initiating HSCs that persist and expand through conventional therapies and are major contributors to disease progression^1–4^. In the last few years, single-cell technologies coupled with mouse functional studies have greatly improved our understanding of the molecular mechanisms driving MDS pathogenesis. These studies have revealed that MDS are driven by multistep processes that affect a recurrent set of genes or cytogenetic aberrations, which leads to the clonal expansion of mutant HSCs over their normal counterparts^5^.

The current standard of care for patients with MDS remains the therapy with HMAs. Although HMA therapy results in some clinical improvement in over 60% of patients, the disease eventually becomes resistant to these agents and progresses to secondary acute myeloid leukemia (sAML). Patients who progress to sAML have a median survival duration of only 4–6 months^6,7^.

Advances in sequencing technologies have provided insights into the genetic mechanisms that contribute to the progression of MDS to sAML^8–11^. Aberrant MDS cells that reside in the immunophenotypically defined HSC compartment are the source of disease progression^11^, but how these cells contribute to therapy failure and disease evolution remains largely unknown. This gap in understanding is delaying the development of predictive biomarkers of clinical relapse and the design of second-line therapies for MDS that could greatly benefit patients in whom HMA-based therapy has failed.

Here, we sought to dissect the biological mechanisms that drive HMA therapy failure at the stem-cell level to uncover vulnerabilities in the disease and halt its evolution.

## Results

### Myelodysplastic syndrome stem cells are in two distinct differentiation states

**Fig. 1:**
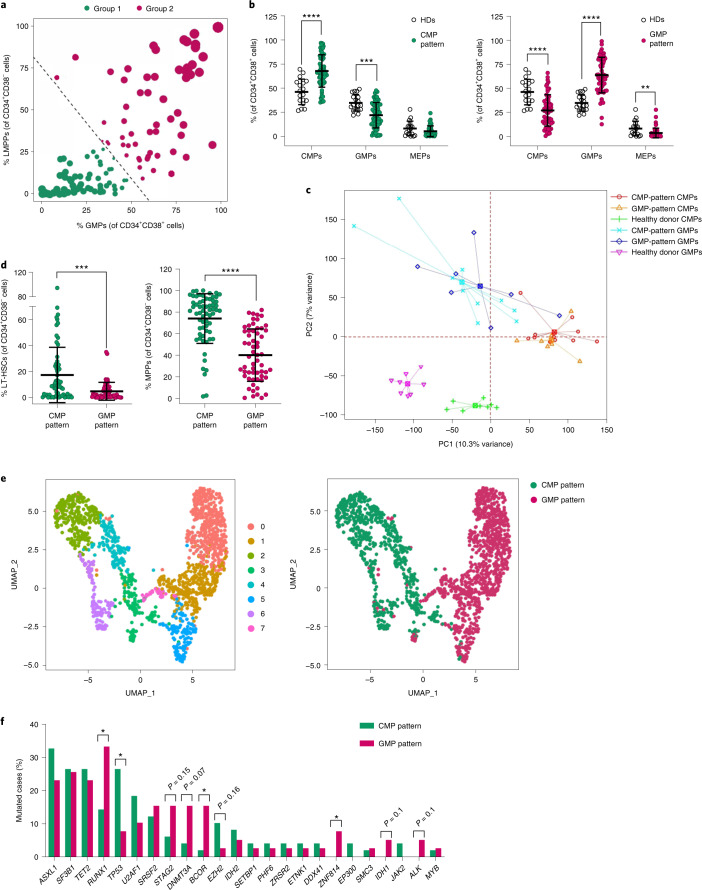
Myelodysplastic syndrome stem cells are in two distinct differentiation states. **a**, Logistic regression stratification of 123 BM samples isolated from untreated patients with MDS based on the frequencies of immunophenotypic LMPPs and GMPs in the lineage-negative (Lin^−^) HSC (CD34^+^CD38^−^) and MyHPC (CD34^+^CD38^+^) compartments, respectively. Each symbol represents one sample. The size of each symbol and its distance from the dashed line are proportional to the sample’s odds of belonging to the specific group. **b**, Frequencies of immunophenotypic CMPs, GMPs, and MEPs in the MyHPC compartment from baseline CMP-pattern MDS samples (left; *n* = 64) and GMP-pattern MDS samples (right; *n* = 59) compared with those from HDs (n = 18). Lines represent means ± s.d. Statistical significance was determined using two-tailed Student *t*-tests (CMP pattern: ****P = 0.000003, ***P = 0.000184; GMP pattern: ****P = 0.000022 and *P* < 0.000001, respectively, **P = 0.0039). **c**, PCA of RNA-seq data from immunophenotypic CMPs and GMPs isolated from the BMs of CMP-pattern patients with MDS (*n* = 11 and *n* = 10, respectively), GMP-pattern patients with MDS (*n* = 7), and HDs (*n* = 8). Each symbol represents one sample. **d**, Frequencies of immunophenotypic LT-HSCs (left) and MPPs (right) in the HSC compartment in CMP-pattern (*n* = 64) and GMP-pattern (*n* = 59) MDS samples. Lines represent medians ± interquartile ranges (IQRs). Statistical significance was calculated using two-tailed Mann–Whitney tests (***P = 0.0001, ****P < 0.0001). **e**, Uniform manifold approximation and projection (UMAP) plots of scRNA-seq data displaying 702 and 970 Lin^−^CD34^+^ cells isolated from representative baseline CMP-pattern and GMP-pattern MDS samples, respectively. Each symbol represents one cell. Different colors represent gene expression cluster types (left) and sample identities (right). **f**, Prevalence of somatic mutations in oncogenes and leukemia-relevant genes in BM MNCs from untreated CMP-pattern (*n* = 49) and GMP-pattern (*n* = 39) MDS samples. Genes mutated in ≥2 patients are shown. Statistical significance was calculated using Chi-squared tests (*RUNX1*, *P = 0.034; *TP53*, *P = 0.023; *BCOR*, *P = 0.022; *ZNF814*, *P = 0.048). Source data

**Extended Data Fig. 1:**
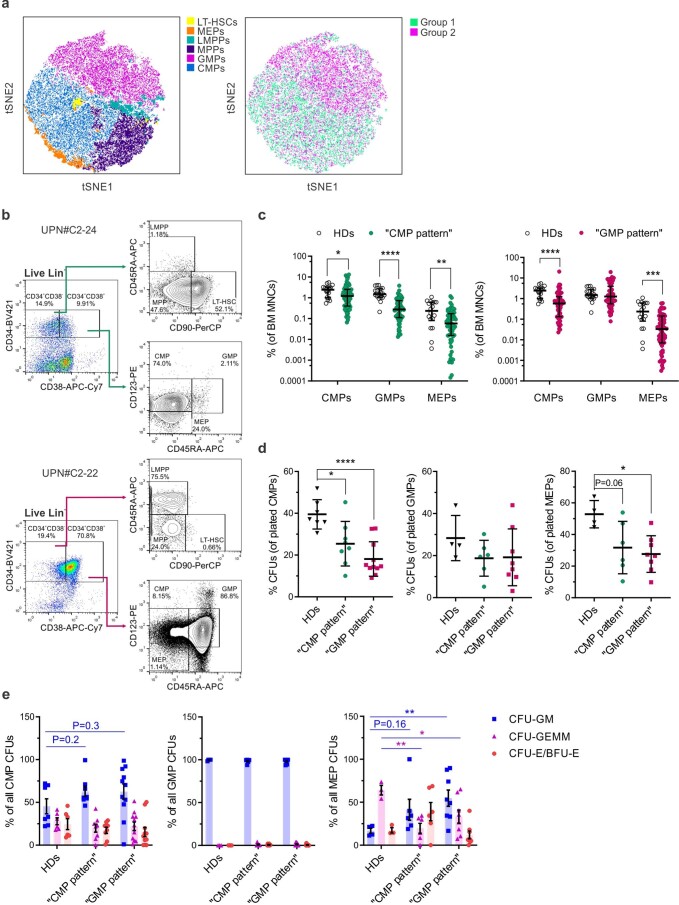
MDS stem cells are in two distinct differentiation states. **(a)** tSNE maps displaying 5,000 Lin^-^CD34^+^ cells from the BM of 122 untreated MDS patients, clustered based on their raw immunophenotypic profiles. Overlaid colors represent the HSPC populations assigned to each cell (left), based on the gating strategy displayed in (b), and the phenotypic group of the corresponding sample of origin of each cell (right). **(b)** Flow cytometry plots of Lin^-^CD34^+^CD38^-^ and Lin^-^CD34^+^CD38^+^ populations in representative CMP-pattern (top) and GMP-pattern (bottom) MDS samples. **(c)** Frequencies of immunophenotypic MyHPC populations in total BM MNCs from CMP-pattern MDS (left; *n*=64) and GMP-pattern MDS (right; *n*=59) samples, compared with those in BM MNCs from HD samples (*n*=18). Lines represent medians ± IQRs. Statistically significant differences between pairs were detected using two-tailed Mann-Whitney tests (CMP-pattern: *P=0.029, ****P<0.0001, **P=0.003; GMP-pattern: ****P<0.0001, ***P=0.0002). **(d)** Plating efficiency of colony-forming units (CFUs) derived from CMPs (left), GMPs (center), and MEPs (right) isolated from the BMs of CMP-pattern MDS (CMPs, *n*=8; GMPs and MEPs, *n*=6) and GMP-pattern MDS (CMPs, *n*=11; GMPs and MEPs, *n*=8), compared with those of HDs (CMPs, *n*=7; GMPs and MEPs, *n*=4). Each symbol represents the average of the technical duplicate; lines represent means ± s.d. Statistical significance was calculated using one-way ANOVA and Tukey’s multiple comparisons test (CMPs: ****P=0.0001, *P=0.014; MEPs: *P*=0.017). **(e)** Frequencies of CFUs derived from CMPs (left), GMPs (center), and MEPs (right) isolated from CMP-pattern MDS (CMPs, *n*=8; GMPs and MEPs, *n*=6) and GMP-pattern MDS (CMPs, *n*=11; GMPs and MEPs, *n*=8) samples, compared with those derived from cells isolated from HDs (CMPs: *n*=6 BFUs-E and GEMMs, *n*=7 GMs; GMPs and MEPs, *n*=4). Lines represent means ± SEMs. Statistical significance was calculated using a mixed model analysis with the Geisser-Greenhouse correction for matched values, using type 3 tests of fixed effects (disease x colony lineage in CMPs: F[4, 67]=1.46, *P=*0.22; GMPs: F[4, 43]=0.97, *P=*0.43; MEPs: F[4, 43])=5.94, *P*=0.0007), and Dunnett’s multiple comparison test (CFU-GM: **P=0.0084; CFU-GEMM: **P=0.0028, *P=0.019). BFU-E, burst forming unit-erythroid; GEMM, granulocyte/erythrocyte/monocyte/megakaryocyte; GM, granulocyte/macrophage. Source data

To identify predictive biomarkers of MDS evolution, we first sought to characterize the immunophenotypic profile of the hematopoietic stem and progenitor cell (HSPC) compartment in a cohort of 123 bone marrow (BM) samples isolated from untreated patients with MDS. Unsupervised hierarchical clustering based on the frequency of immunophenotypically defined HSPC populations^1,12^ (Supplementary Table 1) followed by principal-component analysis (PCA) identified the frequencies of the lymphoid-primed multipotent progenitor (LMPP) and granulocytic–monocytic progenitor (GMP) populations as the main sources of variation across the samples (Supplementary Fig. 1a). Logistic regression analysis yielded the equation log(odds) = 0.5567 × %GMP + 0.4198 × %LMPP − 32.004, which we used to systematically stratify the MDS samples into two main groups (Fig. 1a) independently of International Prognostic Scoring System stratification and World Health Organization classification (Supplementary Table 2). Differences in the cellular composition of the groups’ HSPC compartment were confirmed by *t*-distributed stochastic neighbor embedding (*t*-SNE) analysis (Extended Data Fig. 1a). Compared with BM samples from healthy donors (HDs), the BM samples of one of the MDS groups (52% of the samples) had an abnormal differentiation pattern characterized by an increased frequency of common myeloid progenitors (CMPs) within the myeloid hematopoietic progenitor cell (MyHPC) compartment (a ‘CMP pattern’ of differentiation; Fig. 1b and Extended Data Fig. 1b). In contrast, the BM samples of the other MDS group (48% of the samples) had a higher frequency of GMPs within the MyHPC compartment (a ‘GMP pattern’ of differentiation; Fig. 1b and Extended Data Fig. 1b). The two MDS differentiation patterns did not result from the expansion of either the CMP or the GMP population but were the consequence of the significant decrease of the frequencies of the other two respective progenitor populations in the BM mononuclear cells (MNCs), namely, GMPs and megakaryocyte erythroid progenitors (MEPs) in CMP-pattern MDS and CMPs and MEPs in GMP-pattern MDS (Extended Data Fig. 1c).

**Extended Data Fig. 2:**
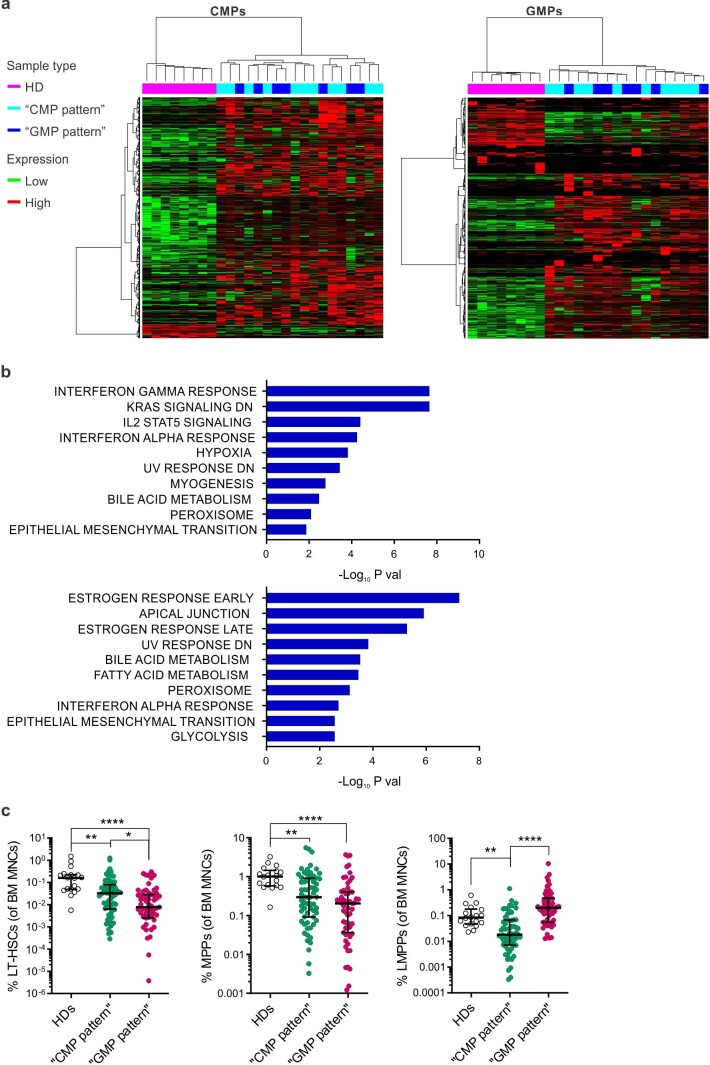
MDS HSPC architectures have myeloid-biased progenitors. **(a)** Unsupervised clustering analysis of genes differentially expressed in CMPs (left; 957 genes) and GMPs (right; 1,369 genes) isolated from untreated CMP-pattern (*n*=11 and *n*=10, respectively) and GMP-pattern MDS patients (*n*=7 in both) and HDs (*n*=8 in both). **(b)** GSEA of genes significantly (*P adj*<0.05) upregulated in CMPs (top; 903 genes) and GMPs (bottom; 951 genes) isolated from MDS patients (*n*=18 and *n*=17, respectively), compared with those from HDs. Hallmark gene sets with a gene enrichment overlap rate (k/K) >0.05 and *P*>0.01 are shown. **(c)** Frequencies of immunophenotypic LT-HSCs (left), MPPs (center), and LMPPs (right) in total BM MNCs samples from HDs (*n*=18), baseline CMP-pattern MDS (*n*=64), and baseline GMP-pattern MDS (*n*=59). Lines represent medians ± IQRs. Statistical significance was calculated using Kruskal-Wallis tests and Dunn’s multiple comparisons test (****P<0.0001, **P<0.01, *P<0.05). Source data

Methylcellulose clonogenic assays of distinct progenitor populations revealed that, compared with HD samples, CMP-pattern MDS and GMP-pattern MDS samples had impaired colony forming (Extended Data Fig. 1d), which is consistent with their defective proliferative and differentiation capacity. Whereas GMP cells isolated from CMP-pattern MDS and GMP-pattern MDS samples retained their overall lineage identities, CMPs and MEPs isolated from the same samples underwent functional reprogramming of their lineage fate toward the myelomonocytic output (Extended Data Fig. 1e). PCA of gene expression signatures in CMPs and GMPs showed that the molecular profiles of these two populations were similar between the two MDS subgroups but substantially different from those of the CMPs and GMPs isolated from HDs (Fig. 1c and Extended Data Fig. 2a). Differential expression analyses of the CMP and GMP populations isolated from MDS samples revealed a significant upregulation of genes involved in interferon gamma signaling (Extended Data Fig. 2b), the constitutive activation of which is associated with impaired myeloid differentiation and apoptosis^13^.

Although CMP-pattern and GMP-pattern progenitor cells had similar functional profiles, the immunophenotypic compositions of their upstream HSC precursors were notably different. Specifically, whereas CMP-pattern MDS samples had higher frequencies of long-term (LT)-HSCs and multipotent progenitors (MPPs; Fig. 1d), GMP-pattern MDS samples had a significantly higher frequency of LMPPs (Fig. 1a). These distinct HSC architectures arose from a significant expansion of the LMPPs in GMP-pattern MDS in the context of an overall decrease in the frequencies of LT-HSCs and MPPs in total BM MNCs (Extended Data Fig. 2c).

**Extended Data Fig. 3:**
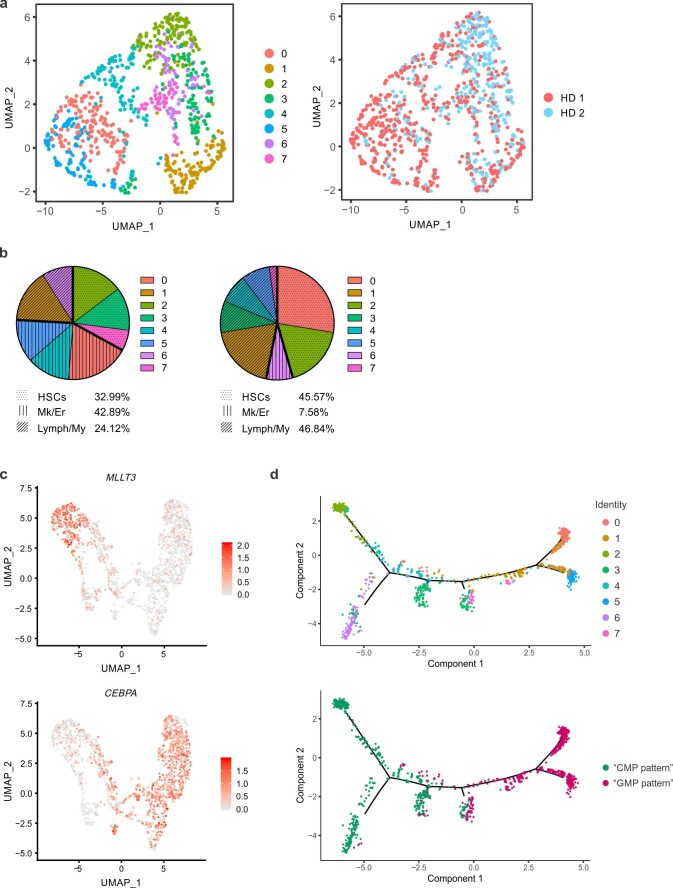
MDS HSPCs are transcriptionally different. **(a)** UMAP plots of scRNA-seq data displaying 878 Lin^-^CD34^+^ cells isolated from the BM samples of two representative HDs. Each symbol represents one cell. Different colors indicate the transcriptional cluster type (left) and sample origin (right) of each cell. **(b)** Distribution of the total number of HD (left) and MDS (right) Lin^-^CD34^+^ cells shown in (a) and in Fig. 1e, respectively, among scRNA-seq clusters with HSC, megakaryocytic/erythroid, or lymphoid/myeloid identity. Lineage identities are represented by different patterns. **(c)** UMAP plots of scRNA-seq data displaying the differential expression of *MLLT3* (top) and *CEBPA* (bottom) in Lin^-^CD34^+^ cells isolated from the representative CMP-pattern and GMP-pattern MDS samples shown in Fig. 1e. Each symbol represents one cell. **(d)** Single-cell trajectory maps of the clusters shown in Fig. 1e. Each symbol represents one cell. Different colors represent transcriptional cluster types (top) and sample identities (bottom). Source data

To determine whether the two MDS groups’ immunophenotypic HSPC architectures were the result of distinct differentiation potentials of the early HSCs or of the aberrant expression of cell surface markers, we analyzed the gene expression profile of the lineage-negative (Lin^−^) CD34^+^ HSPC compartment at the single-cell level. Single-cell RNA-sequencing (scRNA-seq) analysis of HSPCs isolated from HDs and from two representative MDS samples with markedly different immunophenotypic patterns of differentiation (Extended Data Fig. 1b) resulted in cell clusters driven by sample type and the cells’ differentiation potential (Supplementary Fig. 1b,c). Whereas the HSPCs from HD samples had two distinct and continuous erythroid/megakaryocyte and myeloid/lymphoid differentiation trajectories (Extended Data Fig. 3a,b and Supplementary Fig. 2a), which is in line with the current view of hematopoiesis^14,15^, HSPCs from CMP-pattern and GMP-pattern MDS samples had a predominantly myeloid differentiation trajectory (Fig. 1e, Extended Data Fig. 3b and Supplementary Fig. 2b). Differential expression analysis of the two MDS samples showed that the cells atop the HSPC hierarchy in CMP-pattern MDS retained the transcriptional profile of the more immature LT-HSCs, including the expression of the transcription factors encoded by *MLLT3*, *PBX1* and *HLF*. In contrast, the cells atop the HSPC hierarchy in GMP-pattern MDS were characterized by the expression of myeloid-affiliated genes previously identified in the LMPP population^16^, including the early myeloid transcriptional factor encoded by *CEPBA* and the colony-stimulating factor receptor encoded by *CSF3R* (Extended Data Fig. 3c and Supplementary Table 3). Pseudotime analysis^17^ of the HSPCs’ transcriptional dynamics showed that although each group’s earlier hematopoietic cells were in a distinct differentiation state, these cells’ differentiation trajectories converged at the myeloid progenitor state (Extended Data Fig. 3d). These results are consistent with our observation that CMP-pattern and GMP-pattern MyHPC populations display similar myelomonocytic differentiation potentials (Extended Data Fig. 1e) and explain why the clinical phenotypes of the two groups of patients with MDS did not differ much (Supplementary Table 2).

### The two myelodysplastic syndrome architectures are driven by different genetic alterations

**Extended Data Fig. 4:**
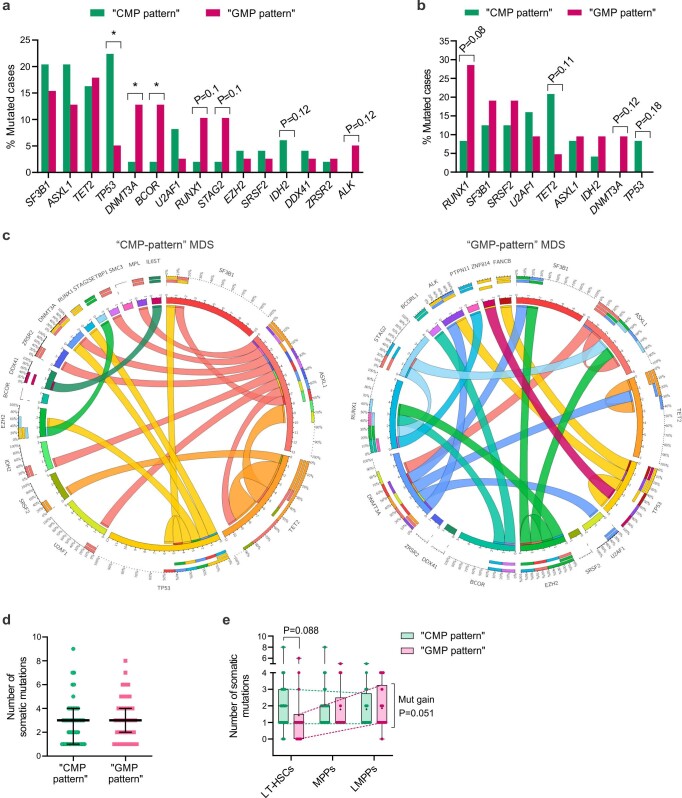
The two MDS architectures are driven by different genetic alterations. **(a)** Prevalence of founding somatic mutations in dominant clones in BM MNCs from untreated CMP-pattern (*n*=49) and GMP-pattern (*n*=39) MDS samples. Genes whose founding mutations were detected in ≥2 patients are shown. Statistical significance was calculated using chi-square tests (*TP53*:*P=0.023; *DNMT3A*: *P=0.046; BCOR: *P=0.046). **(b)** Prevalence of founding somatic mutations in secondary non-branching clones in BM MNCs from untreated CMP-pattern (*n*=24) and GMP-pattern (*n*=21) MDS samples. Genes whose founding mutations were detected in ≥2 patients are shown. Statistical significance was calculated using chi-square tests. **(c)** Co-mutation patterns in dominant BM MNC clones from CMP-pattern (left; *n*=49) and GMP-pattern (right; *n*=39) MDS patients. Founder genes whose mutations were detected in ≥2 patients are shown along with genes secondarily mutated in the same clones. Genes are color-coded by the inner circular segments, whose sizes are proportional to the total numbers of mutations in the cohort. Ribbons connect genes whose mutations co-occur in a dominant clone, color-matching the founder gene (first hit). Outer circular segments represent the relative contribution of mutations in other genes to a gene’s co-mutation profile. Missing outer colored segments represent cases in which the gene was a single-hit founder (no co-mutation). **(d)** Numbers of somatic mutations in BM MNCs from CMP-pattern (*n*=49) and GMP-pattern MDS (*n*=39) patients. Lines represent medians and IQRs. No statistical significance was found by a two-tailed Mann-Whitney test. **(e)** Numbers of somatic mutations in HSC subpopulations from untreated CMP-pattern (*n*=24) and GMP-pattern (*n*=14) MDS patients. Boxes represent medians (center) ± IQRs (maxima and minima); crosses represent means; whisker lines represent ranges. Dotted lines represent mutation gain (Mut gain). Statistical significance was calculated using Wilcoxon rank-sum tests and a parametric analysis as explained in Methods. *P* values are two-tailed. Source data

To evaluate whether the two MDS subtypes are molecularly different, we performed exome sequencing analysis of MNCs and corresponding T cells isolated from the BMs of 88 untreated patients with MDS. We observed that somatic mutations in *TP53* were significantly correlated with the CMP-pattern phenotype, whereas mutations in *RUNX1*, *DNMT3A*, *BCOR* and *STAG2* were enriched in patients with GMP-pattern MDS (Fig. 1f). Analysis of the clonal composition and mutation hierarchies using the PyClone algorithm^18^ revealed that *TP53*, *DNMT3A*, *BCOR* and *STAG2* were mostly founder mutations in the corresponding MDS subgroup, and *RUNX1* frequently occurred as a secondary hit in dominant clones driven by *BCOR* and *STAG2* mutations (Extended Data Fig. 4a–c). Other mutations that were not associated with either of the two MDS hierarchies as founder mutations, such as *ASXL1* and *TET2* mutations, showed specific patterns of mutational co-occurrence in the two groups (Extended Data Fig. 4c), which suggests that not only driver mutations but also their combinations with specific secondary hits drive the two MDS phenotypes. Moreover, exome sequencing analysis of LT-HSCs, MPPs and LMPPs isolated from 46 of these 88 MDS samples revealed that, whereas the two groups had similar numbers of somatic mutations in MNCs (Extended Data Fig. 4d), LT-HSCs from CMP-pattern MDS samples accumulated more mutations than did those from GMP-pattern MDS samples, in which the acquisition of the mutational burden occurred stepwise in hierarchically different HSC subpopulations (Extended Data Fig. 4e). Interestingly, in one CMP-pattern MDS sample (17-20) and one GMP-pattern MDS sample (1-248), each with a normal karyotype (Supplementary Table 4), the somatic mutations arose not in the LT-HSCs but in the MPPs, which suggests that MDS can also originate from the transformation of lineage-primed progenitor precursors (Supplementary Figs. 3 and 4).

Collectively, these results demonstrate that the immunophenotypically defined CMP pattern and GMP pattern of myeloid differentiation are biomarkers of distinct transcriptional states and differentiation trajectories of the early MDS HSCs. These routes of differentiation between the two groups are driven by both single founder mutations (or these mutations’ cooperative effect with specific concurrently secondary mutations) and the number of mutations affecting different HSC populations.

### Myelodysplastic syndrome hematopoietic stem cells maintain the disease phenotype in patients with myelodysplastic syndrome and myelodysplastic syndrome-like mice during hypomethylating agent therapy

**Fig. 2:**
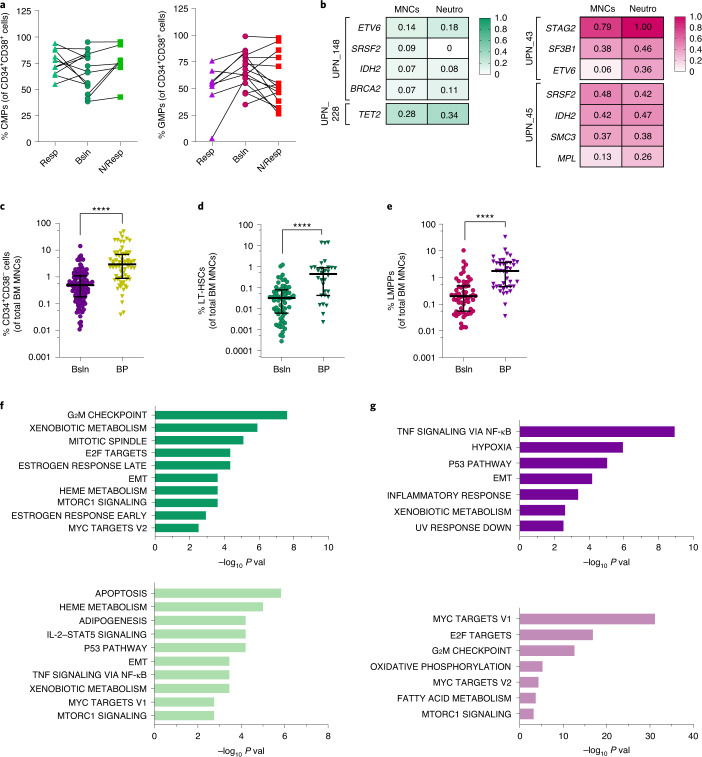
Stem cells maintain the myelodysplastic syndrome phenotype and clonal burden during hypomethylating agent therapy, expand and activate specific survival pathways during blast progression. **a**, Frequencies of CMPs and GMPs in the MyHPC compartment of CMP-pattern and GMP-pattern MDS samples, respectively, sequentially collected at baseline (Bsln; *n* = 15 and *n* = 21) and during HMA therapy at the times of best response (Resp; *n* = 10 and *n* = 8) or no response (N/Resp; *n* = 7 and *n* = 15). No significant differences were detected using one-way analysis of variance (ANOVA) with Dunnett’s multiple-comparisons test (CMPs) and a Kruskal–Wallis test with Dunn’s multiple-comparisons test. **b**, Variant allele frequencies (VAFs) of somatic mutations detected in total BM MNCs and neutrophils (Neutro) from CMP-pattern (left) and GMP-pattern (right) patients with MDS during hematological response to HMAs. **c**, HSC frequencies in total BM MNCs from MDS samples obtained at baseline (*n* = 123) and after HMA failure and BP (*n* = 70). Lines represent medians ± IQRs. Statistical significance was calculated using the two-tailed Mann–Whitney test (****P < 0.0001). **d**, LT-HSC frequencies in total BM MNCs from CMP-pattern MDS samples obtained at baseline (*n* = 64) and after HMA failure and BP (*n* = 30). Lines represent medians ± IQRs. Statistical significance was calculated using the two-tailed Mann–Whitney test (****P < 0.0001). **e**, LMPP frequencies in total BM MNCs from GMP-pattern MDS samples obtained at baseline (*n* = 59) and after HMA failure and BP (*n* = 40). Lines represent medians ± IQRs. Statistical significance was calculated using the two-tailed Mann–Whitney test (****P < 0.0001). **f**, Gene-set enrichment analysis (GSEA) of genes significantly (*P* < 0.004) upregulated (top; *n* = 515) or downregulated (bottom; *n* = 418) in LT-HSCs isolated from CMP-pattern MDS patients with BP (*n* = 4) compared with those from patients at baseline (*n* = 5). Hallmark gene sets with a gene enrichment overlap rate (k/K) > 0.02 and *P* < 0.01 are shown. EMT, epithelial-mesenchymal transition. **g**, GSEA of genes significantly (*P* < 0.01) upregulated (top; *n* = 352) or downregulated (bottom; *n* = 164) in LMPPs isolated from GMP-pattern MDS patients with BP (*n* = 6) compared with those from patients at baseline (*n* = 6). Hallmark gene sets are shown (*P* < 0.01; k/K > 0.02). Source data

**Extended Data Fig. 5:**
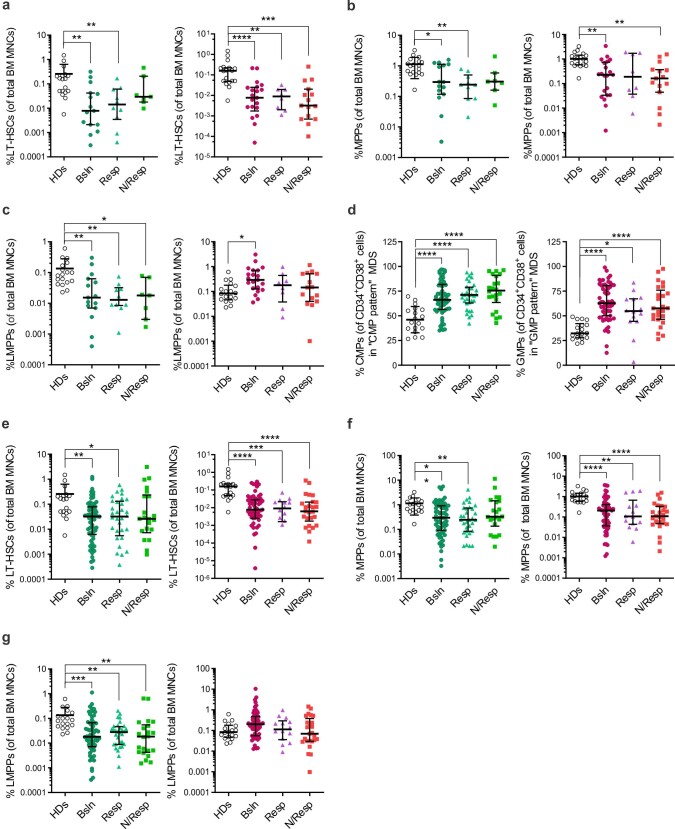
MDS HSCs maintain the disease phenotype in MDS patients during HMA therapy. **(a-c)** Frequencies of immunophenotypic LT-HSCs (a), MPPs (b) and LMPPs (c) in BM MNCs from samples sequentially collected from CMP-pattern (left) and GMP-pattern (right) MDS patients at baseline (Bsln; *n*=15 and *n*=21, respectively) and during HMA therapy at the time of best response (Resp; *n*=10 and *n*=8, respectively) and/or lack of response (N/Resp; *n*=7 and *n*=15, respectively), compared with those of HDs (*n*=18). Lines represent medians ± IQRs. Statistical significance was calculated using Kruskal-Wallis tests and Dunn’s multiple comparison tests (****P<0.0001, ***P<0.001, **P<0.01, *P<0.05). **(d)** Frequencies of immunophenotypic CMPs and GMPs in the MyHPC compartment of the total cohort of CMP-pattern (left) and GMP-pattern (right) MDS patients, respectively, compared with those of HDs (*n*=18). MDS samples were collected at baseline (*n*=64 and *n*=59, respectively) and during HMA therapy, at the time of best available response (*n*=32 and *n*=14, respectively) or primary lack of response (*n*=22 and *n*=25, respectively). Lines represent means ± s.d. Statistical significance was calculated using one-way ANOVA and Tukey’s multiple comparisons tests (****P<0.0001, *P<0.05). **(e-g)** Frequencies of immunophenotypic LT-HSCs (e), MPPs (f) and LMPPs (g) in total BM MNCs from the total cohort of CMP-pattern (left) and GMP-pattern (right) MDS patients at baseline (*n*=64 and *n*=59, respectively) and during HMA therapy at the time of best available response (*n*=32 and *n*=14, respectively) and primary lack of response (*n*=22 and *n*=25, respectively), compared with those of HDs (*n*=18). Lines represent medians ± IQRs. Statistical significance was calculated using Kruskal-Wallis tests and Dunn’s multiple comparisons tests (****P<0.0001, ***P<0.001, **P<0.01, *P<0.05). Source data

Next, to elucidate the effect of HMA-based therapy on the aberrant differentiation architectures of the two MDS groups, we tracked the dynamic changes of the HSPC subpopulations in sequential samples obtained from 15 CMP-pattern and 21 GMP-pattern patients with MDS who were enrolled in clinical trials of single-HMA-based therapy. We observed that aberrant differentiation patterns involving the HSPC compartment persisted during treatment regardless of the hematopoietic hierarchy type or the degree of hematological response (Fig. 2a, Extended Data Fig. 5a–c and Supplementary Fig. 5a,b). Similar results were obtained when we analyzed nonsequential MDS samples (Extended Data Fig. 5d–g). On the basis of these results, which are consistent with our and others’ previous findings showing that patients who achieve hematological response have persistent tumor burden in both BM cells^19^ and end-stage differentiated myeloid cells (Fig. 2b), we hypothesized that disease remission (that is, normalization of the peripheral blood (PB) counts and BM blast number) does not depend on the rescue of differentiation defects at the HSC level but rather results from the HMA-induced hematopoietic differentiation of HSPC-derived genetically abnormal cells.

**Extended Data Fig. 6:**
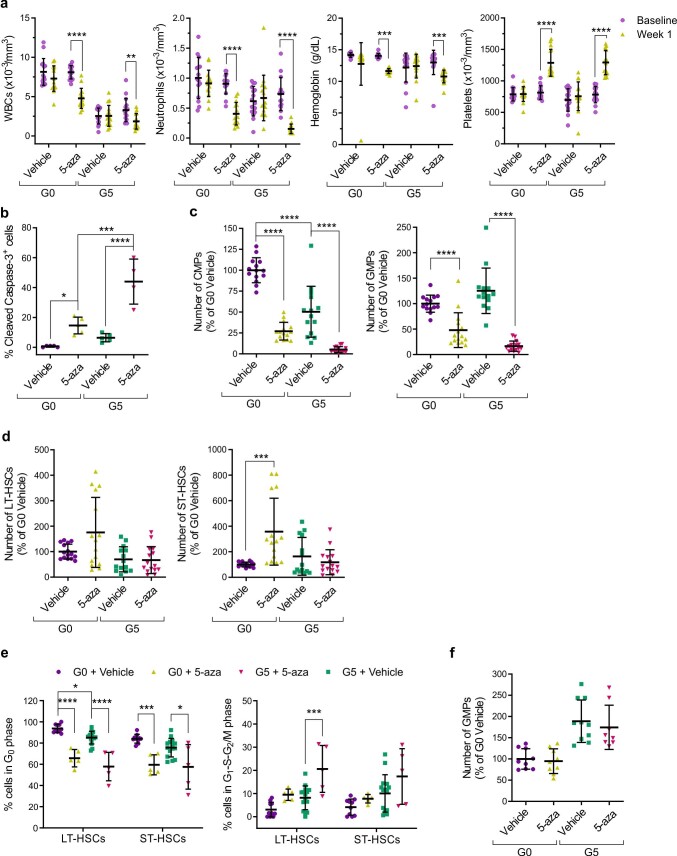
MDS HSCs maintain the disease phenotype in MDS-like mice during HMA therapy. **(a)** Complete blood counts before treatment and after 1 week of treatment with 5-aza or vehicle in G0 (*Tert*^*ER*/+^; *n*=15/each) and G5 (*Tert*^*ER/ER*^; *n*=16/each) mice. Lines represent the means ± s.d. of three independent experiments. Statistical significance was calculated using two-tailed Student t-tests (WBCs: ****P<0.000001, **P=0.0025; Neutrophils: ****P<0.000001; Hemoglobin [left to right]: ***P=0.00034, ***P=0.0006); Platelets: *P*<0.000001). **(b)** Frequency of cleaved caspase-3^+^ BM cells after 1 week of treatment with 5-aza or vehicle in G0 (*n*=5/each) and G5 mice (*n*=4 and *n*=5, respectively). Lines represent means ± s.d. Statistical significance was calculated using one-way ANOVA and Tukey’s multiple comparisons test (****P<0.0001, ***P<0.001, *P<0.05). **(c)** Numbers of CMPs (left) and GMPs (right) in the BMs of G0 and G5 mice treated with 5-aza (*n*=15/each) or vehicle (*n*=14/each) for 1 week. Lines represent the means ± s.d. of three independent experiments. Statistical significance was calculated using one-way ANOVA and Tukey’s multiple comparisons tests (****P<0.0001). **(d)** Numbers of LT-HSCs (left) and ST-HSCs (right) in the BMs of G0 and G5 mice treated with 5-aza (*n*=15/each) or vehicle (*n*=15 and *n*=14, respectively) for 1 week. Lines represent the means ± s.d. of three independent experiments. Statistical significance was calculated using one-way ANOVA and Tukey’s multiple comparisons test (***P<0.001). **(e)** Frequencies of quiescent (left) and cycling (right) LT-HSCs and ST-HSCs from G0 and G5 mice after 1 week of treatment with 5-aza (*n*=5/each) or vehicle (*n*=10 and *n*=15, respectively). Lines represent the means ± s.d. of one representative experiment of two. Statistical significance was calculated using one-way ANOVA and Tukey’s multiple comparisons tests (quiescent, LT-HSCs: ****P<0.0001, *P=0.031; ST-HSCs: ***P=0.0008, *P=0.01; cycling, LT-HSCs: ***P=0.0005). **(f)** Numbers of GMPs in the BMs of G0 and G5 mice treated with two cycles of 5-aza (*n*=9 and *n*=8, respectively) or vehicle (*n*=9 and *n*=10, respectively). Lines represent the means ± s.d. of two experiments. No statistical significance between vehicle- and 5-aza-treated mice was detected using one-way ANOVA and Tukey’s multiple comparisons test. Source data

To test this hypothesis, we cataloged the cellular responses to HMA-based therapy in various hematopoietic populations and assessed these populations’ potential roles in the maintenance of the MDS phenotype and mutational clonal burden. To evaluate whether HMA therapy could overcome aberrant HSPC differentiation, we used the *Tert*^*ER/ER*^ telomerase-deficient mouse model, which recapitulates many of the features of the aberrant myeloid differentiation phenotype observed in patients with MDS^20^ independent of any specific genetic alterations. Treatment with the HMA 5-azacitidine (5-aza) at 2.5 mg per kilogram body weight per day for 7 consecutive days induced significant cytopenia, a decline in neutrophils, a slight decrease in hemoglobin levels and moderate thrombocytosis in *Tert*^*ER/ER*^ mice with short telomeres at generation 5 of crossing (G5) and in their corresponding controls with intact telomeres (G0; Extended Data Fig. 6a). BM hypocellularity (Supplementary Fig. 6a) and increased apoptosis (Extended Data Fig. 6b and Supplementary Fig. 6b) were consistent with the cytotoxic effects HMA therapy has on differentiated hematopoietic cells. However, immunophenotypic quantification of the HSPC populations in the BM of the 5-aza-treated mice (Supplementary Fig. 6c) revealed that whereas Lin^−^Sca1^−^c-Kit^+^CD34^+^CD16/32^−^ CMPs and Lin^−^Sca1^−^c-Kit^+^CD34^+^CD16/32^+^ GMPs were significantly depleted after HMA treatment (Extended Data Fig. 6c), the more primitive Lin^−^Sca1^+^c-Kit^+^CD34^−^Flk2^−^ LT-HSCs and Lin^−^Sca1^+^c-Kit^+^CD34^+^Flk2^−^ short-term (ST)-HSCs survived HMA treatment (Extended Data Fig. 6d) and entered the cell cycle to aberrantly reconstitute the downstream progenitors (Extended Data Fig. 6e and Supplementary Fig. 6d). Consistent with this observation, skewed myeloid differentiation was restored in the BM of mice treated with two cycles of therapy (Extended Data Fig. 6f) that mirrored the 1-week-on, 3-weeks-off HMA treatment schedule used in clinical trials (Supplementary Fig. 6e).

**Extended Data Fig. 7:**
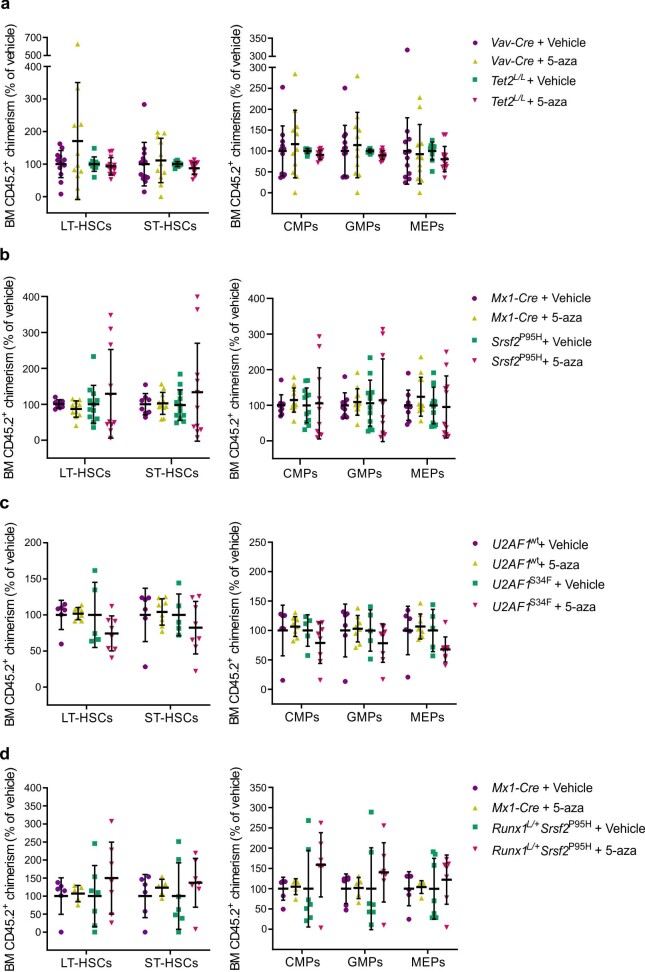
MDS HSCs maintain the BM clonal burden in MDS-like mice during HMA therapy. **(a)** Normalized CD45.2^+^ chimerism rates in HSCs (left) and MyHPCs (right) from mice competitively transplanted with CD45.1^+^ WT and CD45.2^+^ *Vav-Cre* or *Vav-Cre/Tet2*^*L/L*^ (*Tet2*^*L/L*^) cells after two cycles of treatment with 5-aza (*n*=11 and *n*=13, respectively) or vehicle (*n*=13 and *n*=10, respectively). Lines represent the means ± s.d. of two independent experiments. No statistically significant differences were detected by one-way ANOVAs and Tukey’s multiple comparison tests for each cell type. **(b)** Normalized CD45.2^+^ chimerism frequencies in HSCs (left) and MyHPCs (right) from mice competitively transplanted with CD45.1^+^ WT and CD45.2^+^ *Mx1-Cre* or *Mx1-Cre/Srsf2*^*P95H*^ (*Srsf2*^*P95H*^) cells after two cycles of treatment with 5-aza (*n*=12 and *n*=11, respectively) or vehicle (*n*=9 and *n*=12, respectively). Lines represent the means ± s.d. of two independent experiments. No statistically significant differences were detected using one-way ANOVAs and Tukey’s multiple comparison tests for each cell type. **(c)** Normalized CD45.2^+^ chimerism frequencies in HSCs (left) and MyHPCs (right) from mice competitively transplanted with CD45.1^+^ WT and CD45.2^+^ *U2AF1*^wt^/*rtTA* or *U2AF1*^*S34F*^*/rtTA* cells after two cycles of treatment with 5-aza (*n*=8/each) or vehicle (*n*=6 and *n*=5, respectively) in the presence of doxycycline. Lines represent the means ± s.d. of one experiment. No statistically significant differences were detected using one-way ANOVAs and Tukey’s multiple comparison tests for each cell type. **(d)** Normalized CD45.2^+^ chimerism frequencies in HSCs (left) and MyHPCs (right) from mice competitively transplanted with CD45.1^+^ WT and CD45.2^+^ *Mx1-Cre* or *Mx1-Cre/Runx1*^*L/+*^*/Srsf2*^*P95H*^ (*Runx1*^*L/+*^*/Srsf2*^*P95H*^) cells after two cycles of treatment with 5-aza (*n*=5 and *n*=7, respectively) or vehicle (*n*=6 and *n*=7, respectively). Lines represent the means ± s.d. of one experiment. No statistically significant differences were detected using one-way ANOVAs and Tukey’s multiple comparison tests for each cell type. Source data

To evaluate whether HMA therapy can decrease the mutational burden in the HSPC populations, we generated different chimeric mouse models carrying four of the most frequent founder mutations identified in MDS (Extended Data Fig. 4a) by competitively transplanting lethally irradiated CD45.1^+^ wild-type (WT) recipient mice with CD45.2^+^ *Vav1 (Vav)-Cre/Tet2*^*L/L*^, *Mx1-Cre/Srsf2*^*P95H*^, *U2AF1*^*S34F*^*/rtTA* or *Mx1-Cre/Runx1*^*L/L*^*/Srsf2*^*P95H*^ cells and CD45.1^+^ WT BM cells (Supplementary Fig. 6f). We observed that the frequencies of chimerism in the HSC and MyHPC populations (Extended Data Fig. 7a–d) did not change when two cycles of HMA therapy were administered to the chimeras, which suggests that HMA therapy does not affect the mutational burden of the MDS clone. Collectively, these data suggest that MDS stem cells maintain the disease phenotype and clonal burden during HMA therapy and that failure to eradicate these populations can lead to disease relapse and/or leukemic progression.

### Distinct myelodysplastic syndrome hematopoietic stem cells expand during myelodysplastic syndrome progression and activate specific survival pathways

**Extended Data Fig. 8:**
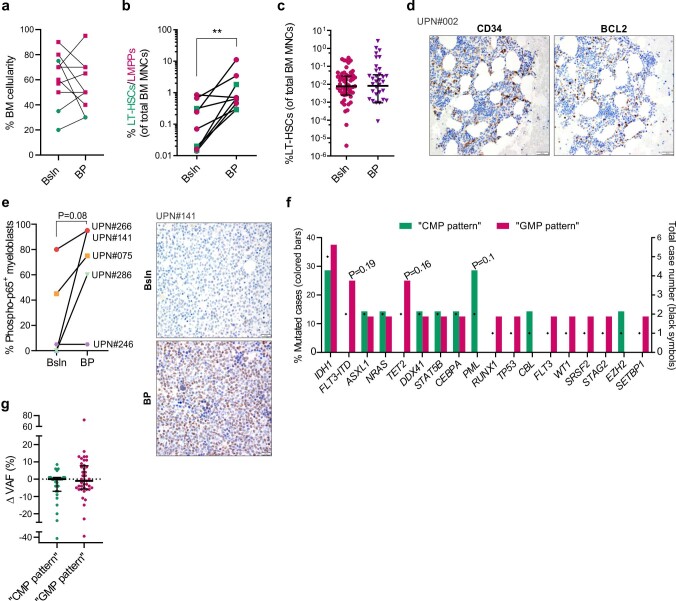
Distinct MDS HSCs expand during MDS progression and activate specific survival pathways. **(a)** Cellularity rates in BM biopsies sequentially collected from CMP-pattern (*n*=3) and GMP-pattern (*n*=7) MDS patients at baseline (Bsln) and at the time of BP. **(b)** Frequencies of immunophenotypic LT-HSCs and LMPPs in total BM MNCs from CMP-pattern (*n*=3) and GMP-pattern (*n*=6) MDS patient samples, respectively, sequentially collected at bsln and BP. Statistical significance was calculated using a two-tailed ratio paired t-test (**P=0.0021). **(c)** Frequencies of immunophenotypic LT-HSCs in total BM MNCs from GMP-pattern MDS samples obtained at bsln (*n*=59) and BP (*n*=40). Lines represent medians ± IQRs. Statistical significance was calculated using the two-tailed Mann-Whitney test. **(d)** CD34 and BCL-2 expression in overlapping BM biopsy specimens obtained from a representative CMP-pattern MDS patient after HMA therapy failure with BP. Scale bars, 20 µm (400x magnification). **(e)** Frequency of phospho-p65^+^ cells in sequential BM biopsy specimens from five GMP-pattern MDS patients at baseline and BP. Statistical significance was calculated using a paired two-tailed Student t-test. A representative phospho-p65 staining is shown on the right. Scale bars, 20 µm (400x magnification). **(f)** Prevalence of newly detected somatic mutations in total BM MNCs from CMP-pattern (*n*=7) and GMP-pattern (*n*=8) MDS patients with BP. No statistically significant differences were detected using chi-square tests. **(g)** Variation in the VAFs of somatic mutations detected in BM MNCs from CMP-pattern and GMP-pattern MDS patients (*n*=11/each) at BP, compared with baseline. Each dot represents one variant (*n*=30 and *n*=40, respectively). Lines represent medians ± IQRs. No statistically significant differences were detected using a two-tailed Mann-Whitney test. Source data

Accordingly, blast progression (BP) following HMA therapy failure in patients with MDS invariably coincided with the expansion of the CD34^+^CD38^−^ HSC compartment (Fig. 2c) in the absence of significant changes in BM cellularity (Extended Data Fig. 8a). However, whereas CMP-pattern MDS with BP was characterized by a significant increase in the total BM MNC frequency of LT-HSCs (Fig. 2d, Extended Data Fig. 8b and Supplementary Fig. 7a), GMP-pattern MDS with BP was characterized by the expansion of LMPPs (Fig. 2e, Extended Data Fig. 8b and Supplementary Fig. 7b) without any significant change in the number of LT-HSCs (Extended Data Fig. 8c).

Then, we sought to elucidate the biological mechanisms underpinning BP in the two MDS groups, as such an understanding might lead to the development of new therapeutic approaches to prevent or overcome HMA failure. Given that HMA failure is mostly independent of the molecular and genetic alterations in the founder clone^21^ and that BP is mostly associated with the expansion of HSC clones carrying preexisting or newly acquired recurrent mutations in genes involved in signal transduction and transcriptional and epigenetic regulation^9,10,22^, we hypothesized that HSC expansion can be induced by key oncogenic pathways that are recurrently activated in each MDS group. To test this hypothesis, we evaluated gene expression changes in LT-HSC and LMPP populations isolated from CMP-pattern and GMP-pattern MDS patients, respectively, whose disease had become resistant to HMA therapy and progressed to higher-risk disease or sAML.

RNA-seq analysis revealed that, compared with those isolated from untreated patients, LT-HSCs isolated from CMP-pattern MDS patients with BP following HMA therapy failure had significantly upregulated genes involved in promoting cell proliferation and survival, including the anti-apoptotic regulator B cell lymphoma 2 (BCL-2), encoded by *BCL2* (Fig. 2f and Supplementary Table 5). Importantly, transgenic mice that overexpress BCL-2 in the HSC compartment are characterized by an important increase in LT-HSCs and repopulation capabilities^23^, which suggests that the BCL-2-induced inhibition of the apoptosis of LT-HSCs is, per se, sufficient to promote aberrant HSC expansion. That BCL-2 was also highly expressed in the CD34^+^ blastic population during disease progression was confirmed by immunohistochemistry (IHC) of BM biopsy specimens (mean of positive cells, 70% ± 11.73%; *n* = 5; Extended Data Fig. 8d). Collectively, these findings suggest that CMP-pattern MDS patients with BP can benefit from treatment with the highly selective BCL-2 inhibitor ABT-199 (venetoclax).

In striking contrast to our findings in CMP-pattern MDS, genes involved in the tumor necrosis factor (TNF)-induced nuclear factor-kappa B (NF-κB) signaling pathway were significantly upregulated in the LMPPs from GMP-pattern MDS patients with BP as compared with LMPPs from GMP-pattern MDS patients with newly diagnosed disease (Fig. 2g and Supplementary Table 6). Consistent with this observation, the frequencies of phospho-NF-κB/p65^+^ blasts (Extended Data Fig. 8e) significantly increased during BP in about 80% of the BM samples isolated from GMP-pattern MDS patients. This molecular signature of inflammation was associated with the significantly decreased expression of genes involved in the regulation of cell proliferation and mitochondrial respiration (Fig. 2g), which suggests that LMPPs lose their differentiation capability and acquire a protective, stem cell-like quiescent state during disease progression. Interestingly, we did not observe any significant differences in the types of newly detected mutations or in the clonal expansion of preexisting mutations between the two MDS subtypes in a cohort of 23 sequential samples obtained at baseline and BP (Extended Data Fig. 8f,g). It is tempting to speculate that the survival pathways upregulated at BP in the two MDS groups are induced both by the specific mutations present in the two MDS subgroups at baseline (for example, *RUNX1* mutations are known to activate the NF-κB pathway^24^) and by changes in the microenvironment occurring at BP (for example, TNF upregulation in the microenvironment of GMP-pattern MDS further enhances NF-κB pathway activation^25^).

### Pharmacologically targeting the upregulated survival pathways in myelodysplastic syndrome hematopoietic stem cells reduces tumor burden and halts disease progression

**Fig. 3:**
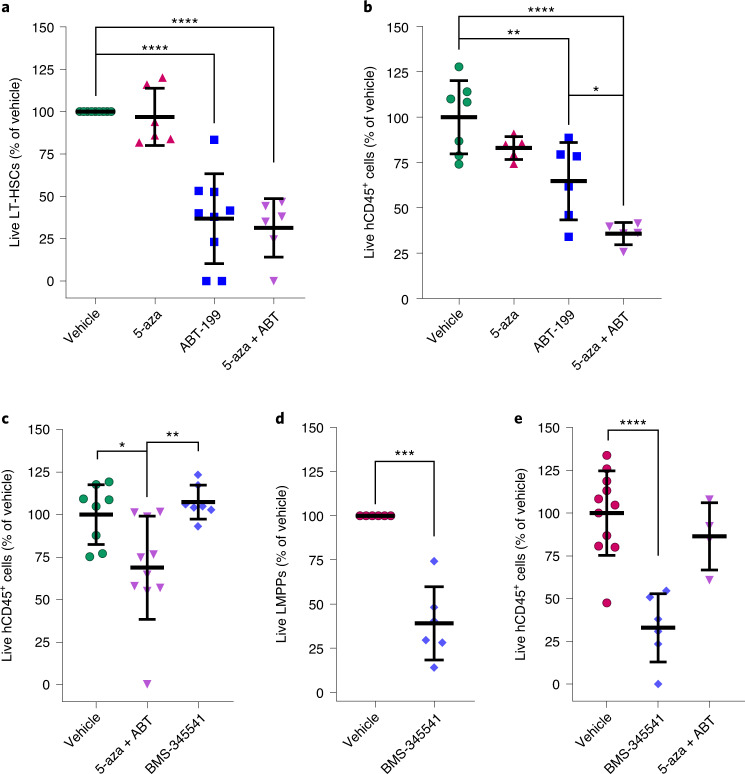
Pharmacologically targeting upregulated survival pathways in myelodysplastic syndrome stem cells reduces tumor burden and halts disease progression. **a**, Numbers of live cultured LT-HSCs from CMP-pattern MDS patients with BP after treatment with 0.5 µM 5-aza (*n* = 6), 50 nM ABT-199 (ABT; *n* = 9) or the combination of the agents (*n* = 6) for 72 h. Lines represent means ± s.d. Statistical significance was calculated using one-way ANOVA and Tukey’s multiple-comparison test (****P < 0.0001). **b**, Human CD45 chimerism in total BM cells from MDS-L xenografts after one cycle of treatment with vehicle (*n* = 7), 5-aza (*n* = 5), ABT-199 (*n* = 6) or the combination of the agents (*n* = 5). Lines represent the means ± s.d. of one experiment. Statistical significance was calculated using one-way ANOVA and Tukey’s multiple-comparison test (****P < 0.0001, **P = 0.0049, *P = 0.038). **c**, Human CD45 chimerism in total BM white blood cells (WBCs) from xenografts of a CMP-pattern BP sample after treatment with vehicle (*n* = 8), 5-aza plus ABT-199 (*n* = 10) or with BMS-345541 (*n* = 7). Lines represent the means ± s.d. of two independent experiments. Statistical significance was calculated using one-way ANOVA and Tukey’s multiple-comparisons test (**P = 0.0056, *P = 0.02). **d**, Numbers of live cultured LMPPs isolated from samples of GMP-pattern MDS with BP (*n* = 6) after treatment with 5 µM BMS-345541 for 48 h in the presence of 2.5 ng ml^−1^ human-recombinant TNF. Lines represent means ± s.d. Statistical significance was calculated using a paired two-tailed Student *t*-test (***P = 0.0008). **e**, Human CD45 chimerism in total BM WBCs from xenografts of a GMP-pattern BP sample after treatment with vehicle (*n* = 11), BMS-345541 (*n* = 6) or with 5-aza plus ABT-199 (*n* = 4). Lines represent the means ± s.d. of three independent experiments. Statistical significance was calculated using one-way ANOVA and Dunnett’s multiple-comparisons test (****P < 0.001). Source data

**Extended Data Fig. 9:**
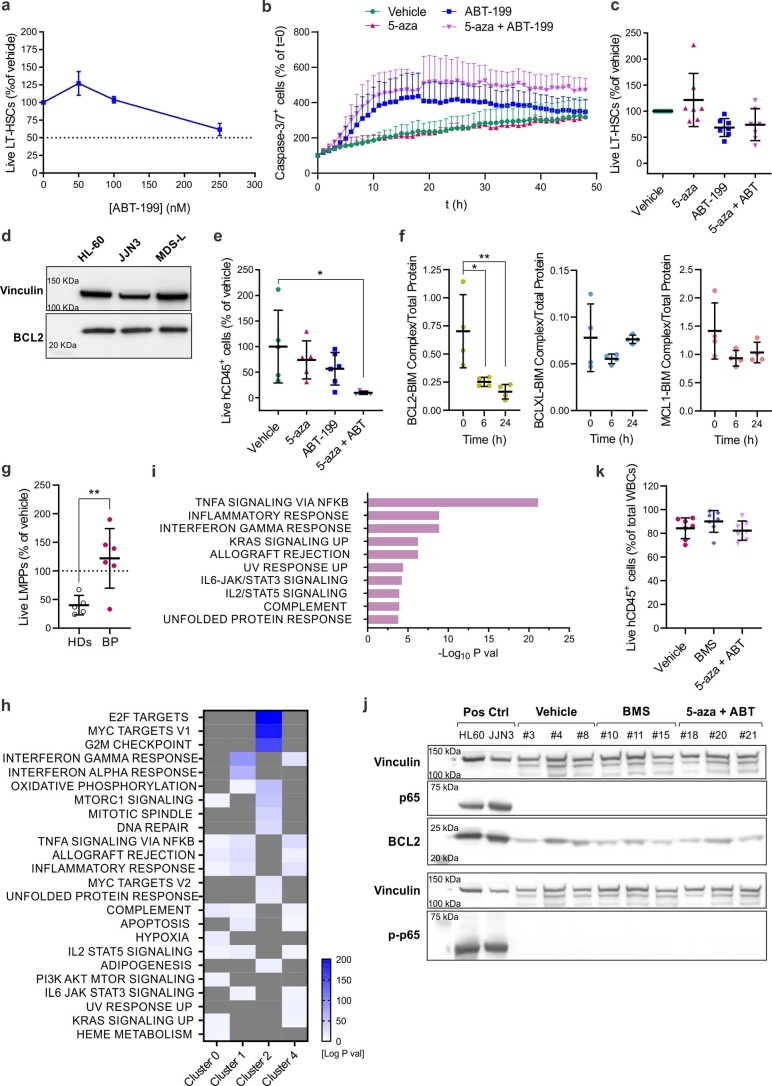
Pharmacologically targeting the upregulated survival pathways in MDS HSCs reduces tumor burden and halts disease progression. **(a)** Numbers of live cultured LT-HSCs from HD BMs (*n*=3) after 72 h of treatment with ABT-199. Lines represent means ± SEMs. **(b)** Numbers of active caspase-3/7^+^ Lin^-^CD34^+^ cells from CMP-pattern MDS patients with BP (*n*=2) treated with 50 nM ABT-199 and/or 0.5 µM 5-aza over 48 h. Symbols and lines represent means + SEMs. **(c)** Numbers of live LT-HSCs from baseline CMP-pattern MDS BM samples (*n*=7) after treatment with 0.5 µM 5-aza and/or 50 nM ABT-199 (ABT) for 72 h. Lines represent means ± s.d. No statistical significance was found using one-way ANOVA and Tukey’s multiple comparison test. **(d)** Representative Western blot of BCL-2 in MDS-L cells. The experiment was performed twice. Molecular weight marker positions are illustrative; a picture of the membrane can be visualized in the corresponding Source Data file. **(e)** Human CD45 chimerism in total splenic WBCs from MDS-L xenografts after treatment with vehicle (*n*=5), 5-aza (*n*=5), ABT-199 (*n*=6), or 5-aza and ABT-199 (*n*=5). Lines represent the means ± s.d. from one experiment. Statistical significance was calculated using one-way ANOVA and Tukey’s multiple comparison tests (*P=0.018). **(f)** Ratios of human BIM complexes with BCL-2, BCL-XL, and MCL-1 in BM cells isolated from MDS-L xenografts treated with ABT-199 for the indicated times (*n*=4/each). Lines represent the means ± s.d. of one experiment. Statistical significance was calculated using one-way ANOVAs and Dunnett’s multiple comparisons tests (**P=0.0062, *P=0.017). **(g)** Numbers of live BM LMPPs from HDs (*n*=5) and GMP-pattern MDS patients with BP (*n*=6) after treatment with 2.5 ng/mL hrTNF for 48 h. Lines represent means ± s.d. Statistical significance was calculated using an unpaired two-tailed Student t-test (**P=0.0087). **(h)** GSEA of genes significantly (*P adj*<0.05) upregulated in human myeloblasts (clusters 0, 1, 2, and 4 in Supplementary Figure 9a). Hallmark gene sets with a *P*<0.0001 and a gene enrichment overlap rate (k/K)>0.02 are shown. **(i)** GSEA of genes significantly (*P adj*<0.05) downregulated in human LMPPs and myeloblasts (clusters 0, 1, 2, and 3 in Supplementary Fig. 9b) from xenografts treated with BMS-345541 compared with those from xenografts treated with vehicle. Hallmark gene sets with a *P*<0.001 and a k/K>0.02 are shown. **(j)** Western blot of p65, phospho-p65 (p-p65), and BCL-2 in human cells isolated from GMP-pattern MDS xenografts from a patient with BP treated with BMS-345541 (BMS) or ABT-199 (ABT) in combination with 5-aza. The experiment was performed once. Molecular weight marker positions are illustrative; a picture of the membrane can be visualized in the corresponding Source Data file. **(k)** Human CD45 chimerism in total BM WBCs from the xenografts described in (j) after treatment. Each symbol represents one sample; lines represent the means ± s.d. of one experiment (*n*=7 mice/group). No statistical significance was detected using one-way ANOVA and Tukey’s multiple comparisons test. Source data

Next, we hypothesized that despite their genetic dissimilarities, the HSCs that expand in each MDS group during BP are addicted to the specific survival pathways that are upregulated in these cells and that the pharmacological inhibition of these pathways induces HSC death, thereby halting MDS progression. As a proof of concept, we isolated HSPCs from CMP-pattern and GMP-pattern MDS patients whose disease progressed despite HMA therapy and treated these cells with specific inhibitors targeting each survival pathway. Three days of treatment with the BCL-2 inhibitor ABT-199 at a dose that did not deplete LT-HSCs isolated from HDs (Extended Data Fig. 9a), either alone or in combination with 5-aza, significantly decreased the number of LT-HSCs isolated from CMP-pattern MDS with BP in co-culture with mesenchymal stromal cells (MSCs; Fig. 3a) by inducing apoptosis (Extended Data Fig. 9b). In contrast, ABT-199 did not significantly affect the survival of LT-HSCs isolated from the BM of patients with newly diagnosed disease (Extended Data Fig. 9c). To evaluate the effect of ABT-199 in vivo, we performed xenograft experiments by transplanting patient-derived blastic MDS-L cells^26^, which have a CMP immunophenotypic profile (Supplementary Fig. 8a) and a high level of BCL-2 expression (Extended Data Fig. 9d), into sublethally irradiated NSGS mice (Supplementary Fig. 8b). Flow cytometry and IHC revealed that, compared with those from the untreated controls, BM (Fig. 3b) and splenic (Extended Data Fig. 9e and Supplementary Fig. 8c) samples from MDS-L xenograft mice treated with one cycle of ABT-199 (100 mg per kilogram body weight per day for 14 d) alone or in combination with 5-aza (0.5 mg per kilogram body weight per day on days 1–7 of a 28-d cycle; Supplementary Fig. 8b) had significantly fewer human CD45^+^ cells, which suggests that this therapy is effective in depleting the blast population. Treatment with ABT-199 efficiently and selectively disrupted BCL-2–BIM complexes in MDS-L cells without affecting BCL-XL–BIM and MCL-1–BIM complexes, confirming target engagement by ABT-199 in the xenografts (Extended Data Fig. 9f). Importantly, significantly reduced numbers of human CD45^+^ cells were also observed in the BM of xenografts developed from T cell-depleted CMP-pattern BM MNCs from a patient with BP after treatment with ABT-199 in combination with 5-aza (Fig. 3c and Supplementary Fig. 8d). In contrast, treatment of the same xenografts with the NF-κB pathway inhibitor BMS-345541 (75 mg per kilogram body weight every other day for 3 weeks) did not induce any significant change in human BM chimerism, which confirms that CMP-pattern MDS at BP do not rely on NF-κB pathway activation to maintain survival.

We then isolated HSPCs from GMP-pattern MDS patients whose disease progressed despite HMA therapy and treated these cells in vitro with BMS-345541 in the presence of TNF. We first observed that TNF alone depleted LMPPs from HDs but not those from GMP-pattern MDS (Extended Data Fig. 9g). However, 2 d of treatment with BMS-345541 in the presence of TNF significantly decreased the number of live GMP-pattern LMPPs in co-culture with MSCs (Fig. 3d). These results are consistent with our hypothesis that GMP-pattern LMPPs at BP rely on NF-κB signaling activation to maintain survival in response to high TNF levels in the BM microenvironment. To evaluate the effect of BMS-345541 in vivo, we generated xenografts by transplanting T cell-depleted BM cells isolated from a GMP-pattern MDS sample with BP into NSGS mice. ScRNA-seq analysis of the engrafted human CD45^+^ cells confirmed that genes involved in the TNF-induced NF-κB signaling pathway were significantly upregulated in the blastic populations (Supplementary Fig. 9a and Extended Data Fig. 9h). Accordingly, in vivo treatment of the xenografts with BMS-345541 significantly inhibited TNF-induced NF-κB signaling activation in blastic cells (Supplementary Fig. 9b and Extended Data Fig. 9i) and reduced BM tumor burden (Fig. 3e). In striking contrast, ABT-199 treatment had no cytotoxic effect on the leukemic cells (Fig. 3e). To exclude the off-target effects of BMS-345541 and further validate that BCL-2 activity drives the survival of CMP-pattern cells but not GMP-pattern MDS cells, we generated xenografts from a patient with GMP-pattern MDS, whose blasts did not show any p65 phosphorylation but showed a high level of BCL-2 expression (Extended Data Fig. 9j). As expected, treatment of the xenografts with BMS-345541 or with ABT-199 plus 5-aza did not reduce tumor burden (Extended Data Fig. 9k). Together, these data provide a rationale for the selective use of BCL-2 and NF-κB pathway inhibitors to treat the majority of CMP-pattern and GMP-pattern patients with MDS, respectively, whose disease has progressed despite HMA therapy, and they reveal a means to improve patient stratification in ongoing clinical trials of venetoclax-based therapy for these patients.

### Venetoclax-based therapy selectively targets hematopoietic stem cells from common myeloid progenitor-pattern myelodysplastic syndrome at blast progression after hypomethylating agent therapy failure

**Fig. 4:**
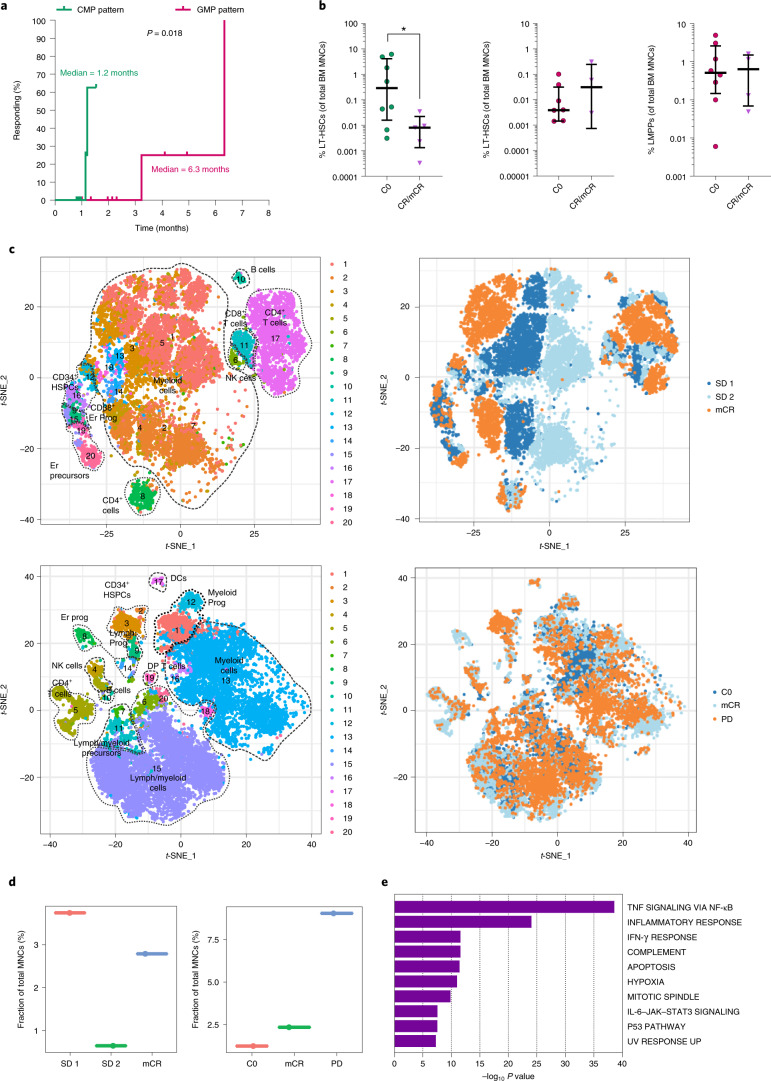
Venetoclax-based-therapy selectively targets hematopoietic stem cells from common myeloid progenitor-pattern myelodysplastic syndrome at blast progression after hypomethylating agent therapy failure. **a**, Cumulative time to achieve CR in CMP-pattern (*n* = 8) and GMP-pattern (*n* = 13) MDS patients with BP after HMA therapy failure treated with venetoclax-based therapy. Statistical significance was calculated using the log-rank (Mantel–Cox) test (*P* = 0.018). **b**, BM MNC frequencies of immunophenotypic LT-HSCs from CMP-pattern MDS patients with BP (left), and LT-HSCs and LMPPs from GMP-pattern MDS patients with BP (center and right). Sequential samples were analyzed before the start of venetoclax-based therapy (C0, cycle 0; *n* = 8 and *n* = 8, respectively) and at the time of hematological remission (CR/mCR, complete remission or marrow CR; *n* = 5 and *n* = 4, respectively). Lines represent means ± IQRs. Statistical significance was determined using two-tailed Mann–Whitney tests (*P = 0.045). **c**, *t*-SNE plots of mass cytometry data displaying 287,354 and 509,697 BM MNCs isolated from a representative patient with CMP-pattern MDS (top) and one patient with GMP-pattern MDS (bottom), respectively, at multiple time points during therapy. Each dot represents one cell. Different colors indicate the hematopoietic clusters (left) and sample origin of each cell (right). Er, erythroid; Prog, progenitors; DCs, dendritic cells; Lymph, lymphoid; DP, double positive (CD4^+^CD8^+^); NK, natural killer; SD, stable disease (SD 1, after 1 cycle of therapy; SD 2, after 2 cycles of therapy); mCR, marrow complete remission; C0, cycle zero; PD, progressive disease. **d**, Frequencies of CD34^+^ HSPCs in the total BM MNC samples from the patients with CMP-pattern MDS (left) and GMP-pattern MDS (right) shown in **c**, at the indicated time points (*n* = 1 sample per time point). **e**, Metascape pathway enrichment analysis of the marker genes of cluster 1 (LMPPs) shown in Supplementary Fig. 11 (adjusted *P* value ≤ 0.05; *n* = 1,722 genes). The top ten Hallmark gene sets are shown. IFN-γ, interferon gamma. Source data

To test the hypothesis that the HSPC architecture can predict the response to venetoclax in a clinical setting, we evaluated the outcomes of a cohort of 21 MDS patients with BP after HMA therapy treated with HMAs and venetoclax at MD Anderson Cancer Center (Supplementary Table 7). Responses were evaluated based on the International Working Group 2003 (ref. ^27^) and 2006 (ref. ^28^) criteria for patients with >20% BM blasts and those with 5–19% BM blasts, respectively, at the time of progression after HMA therapy. Compared with those with GMP-pattern MDS, patients with CMP-pattern MDS had a shorter cumulative time to achieve complete remission (CR; 1.2 months versus 6.5 months; Fig. 4a) and a longer relapse-free survival duration (16.3 months versus 5.2 months; Supplementary Table 7).

**Extended Data Fig. 10:**
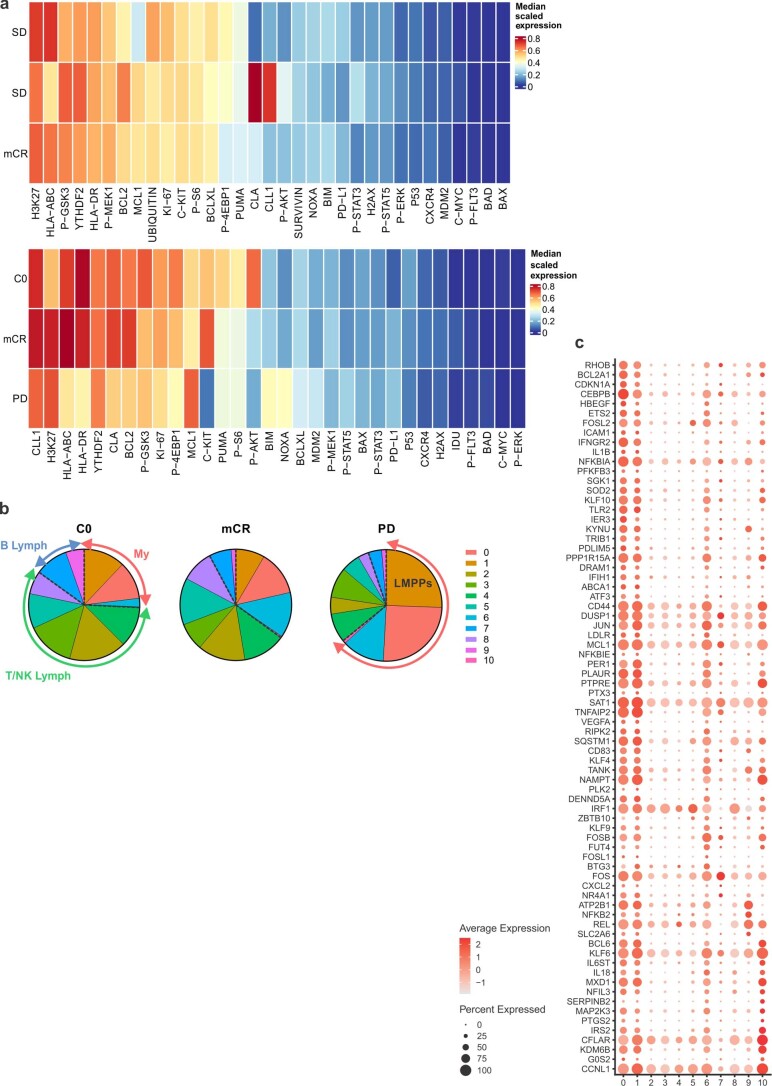
Venetoclax-based therapy selectively targets HSCs from CMP-pattern MDS at BP after HMA therapy failure. **(a)** Heatmaps showing the normalized expression of CyTOF signaling pathway regulators in CD34^+^ HSPC clusters from the sequential CMP-pattern (top) or GMP-pattern (bottom) MDS samples shown in Fig. 4c. SD, stable disease; mCR, marrow complete remission; C0, cycle zero; PD, progressive disease. **(b)** Distribution (%) of the total number of BM MNCs from a GMP-pattern patient with BP among the scRNA-seq clusters shown in Supplementary Fig. 11a at each time point of venetoclax-based therapy. Lymph, lymphoid; My, myeloid. **(c)** Differential expression of cluster 1 marker genes significantly enriched in the TNF signaling via NF-κB pathway as indicated in in Fig. 4e, across all clusters shown in Supplementary Fig. 11. Source data

Consistent with the hypothesis that venetoclax-based therapy selectively targets different HSC populations at BP after HMA failure, we observed a significant decrease in the BM frequencies of LT-HSCs in CMP-pattern MDS but not LT-HSCs or LMPPs in GMP-pattern MDS patients at the time of CR after venetoclax-based therapy (Fig. 4b). To further investigate the changes in the cellular composition induced by venetoclax-based therapy, we used mass cytometry to analyze BM MNCs from patients with CMP-pattern and GMP-pattern MDS at multiple time points during therapy. Our integrated analysis of each patient’s sequential samples identified distinct cellular clusters based on the expression of lineage-specific surface markers (Fig. 4c and Supplementary Fig. 10). Although both CMP-pattern and GMP-pattern MDS CD34^+^ HSPCs expressed high levels of BCL-2 (Extended Data Fig. 10a), venetoclax-based therapy selectively depleted CMP-pattern but not GMP-pattern CD34^+^ HSPCs at the time of CR (Fig. 4d). These data suggest that GMP-pattern MDS HSPCs do not rely on a BCL-2-mediated anti-apoptotic pathway to maintain survival, and they explain why these cells dramatically expanded at the time of BP after venetoclax-based therapy failure (Fig. 4d). Similar results were obtained when we analyzed the same GMP-pattern samples using scRNA-seq (Supplementary Fig. 11 and Extended Data Fig. 10b). Consistent with our preclinical results, this analysis also revealed that HSPCs expanding at BP showed significantly increased expression of genes involved in the TNF-induced NF-κB signaling pathway, including the anti-apoptotic effectors encoded by *MCL1* and *BCL2A1* (Fig. 4e and Extended Data Fig. 10c).

## Discussion

HMA-based therapy remains the only worldwide approved option for patients with MDS. Despite our increasing understanding of the pathophysiology of MDS, the biological mechanisms that drive disease progression after HMA-based therapy remain poorly understood.

Predicting responses or clinical outcomes to HMAs based on MDS patients’ genetic alterations is challenging and results are inconsistent across studies. Indeed, previous analyses of primary samples enrolled in HMA-based clinical trials reported that MDS patients with mutations in epigenetic modifiers such as *TET2* (ref. ^29^) have higher rates of responses to HMA-based therapy, but others could not validate these results^30^. This evidence suggests that genetic alterations alone minimally account for HMA-based therapy outcomes and that, given that MDS are phenotypically heterogeneous, only dissecting the biological properties of the cells of origin for the disease in a large cohort of patients may provide the deeper mechanistic insights into HMA failure to develop new therapeutic strategies and to halt disease progression.

**Fig. 5:**
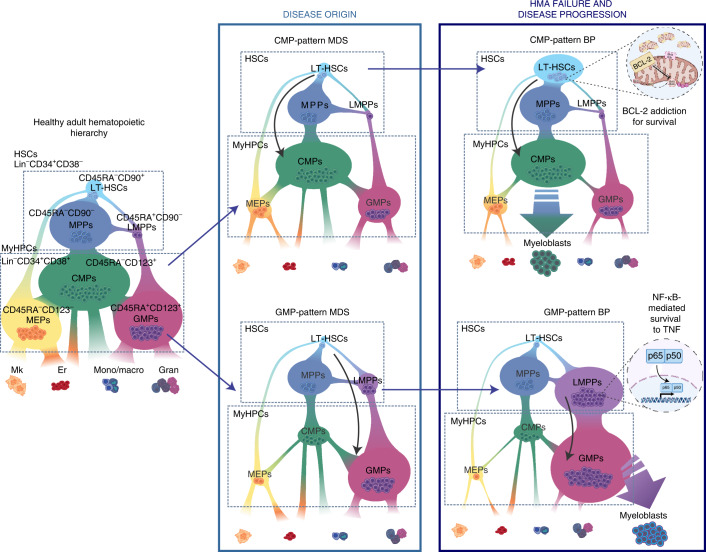
Proposed working model. Distinct differentiation trajectories characterize the HSPC compartment in healthy adults (left) and MDS patients at the time of diagnosis (middle) and progression (right). MDS can be classified as one of two immunophenotypically distinct groups, CMP-pattern MDS or GMP-pattern MDS, based on the frequency of CMPs or GMPs, respectively, in the MyHPC compartment. In each group, MDS stem cells in distinct differentiation states (LT-HSCs in CMP-pattern MDS or LMPPs in GMP-pattern MDS) maintain the disease during HMA-based therapy and expand at progression. Specific survival pathways are selectively activated in each MDS stem cell type at BP and drive disease transformation. Er, erythrocyte; Gran, granulocyte; Mk, megakaryocyte; Mono/macro, monocyte/macrophage.

Here, we provided both preclinical and clinical lines of evidence that MDS is maintained by one of two conserved hierarchically distinct cellular organizations in which cell-type-specific survival pathways drive HMA therapy failure and disease progression (Fig. 5). These data suggest that the cellular architecture of MDS should be considered as a biomarker for predicting the intrinsic vulnerabilities of the cells that expand at relapse and thus for guiding the design or choice of specific therapeutic approaches targeting these cells, particularly in the setting of venetoclax-based therapy. However, other cells in the BM microenvironment, such as immune cells, may play a critical role in mediating the therapeutic outcome of MDS patients, thus opening future questions to address in order to fully understand the disease and prevent or overcome its evolution.

## Methods

### Human primary samples and cell lines

BM specimens from 379 patients diagnosed with MDS and referred to the Department of Leukemia at MD Anderson Cancer Center or the Department of Medicine and Surgery at the University of Parma were obtained with the approval of the institutions’ respective Institutional Review Boards (IRBs) and in accordance with the Declaration of Helsinki. At MD Anderson, BM aspirates were collected under the IRB-approved research protocol PA15-0926. Sequential evaluation of HSPC frequencies and response outcomes to therapy was performed as part of this IRB-approved study. Baseline BM aspirates were collected from patients before any treatment other than supportive care and no less than 1 week after the completion of growth factor therapy. For patients for whom baseline samples were available, sequential BM samples were collected at any time during HMA treatment. For patients already receiving HMA treatment, samples were obtained from cycle 4 onward. All patients received single-HMA therapy, except for 13 patients who simultaneously received the cytidine deaminase inhibitor E7727 (cedazuridine) to increase HMA bioavailability.

BM samples from 21 HDs (median age, 53 years; range, 49–66 years) were obtained from AllCells or from MD Anderson’s Department of Stem Cell Transplantation.

Written informed consent was obtained from all donors, and all MDS diagnoses were confirmed by dedicated hematopathologists. The clinical characteristics of the MDS patients at diagnosis are shown in Supplementary Table 2. Responses to HMA therapy and clinical outcomes are shown in Supplementary Table 8.

BM MNCs were isolated from each sample using the standard gradient separation approach with Ficoll-Paque PLUS (GE Healthcare Lifesciences). For cell sorting, MNCs were enriched in CD34^+^ cells using magnetic sorting with the CD34 Microbead Kit (Miltenyi Biotec) and further purified by FACS. CD3^+^ T cells were positively selected from the CD34^−^ cell fraction using the human CD3 MicroBead Kit (Miltenyi Biotec) and further purified by FACS. Neutrophils were purified by FACS of CD66b^+^ cells (G10F5; 1:20 dilution; BD Biosciences) from BM or PB cells magnetically enriched in CD15^+^ cells using the human CD15 MicroBead Kit (Miltenyi Biotec).

Human BM-derived MSCs were obtained from M. Andreeff’s laboratory after isolation from healthy BM donors as described previously^31^.

The MDS-L cell line was a generous gift of K. Tohyama (Department of Laboratory Medicine, Kawasaki Medical School). The identity of the MDS-L line was confirmed by short tandem repeat DNA fingerprinting at MD Anderson’s Characterized Cell Line Core Facility.

### Clinical data analyses

All untreated patients included in the clinical data analyses were seen at MD Anderson. Clinical data were collected retrospectively by reviewing the patients’ electronic health records. All statistical analyses of clinical data were performed with R statistical software^32^ (v3.5.1). Two-tailed Student *t*-tests or Mann−Whitney tests, where appropriate, and Chi-square tests were used to compare continuous and categorical variables, respectively. Response to HMA therapy was assessed using the 2006 (ref. ^28^) and the 2003 (ref. ^27^) International Working Group response criteria for MDS and sAML, respectively.

### Logistic regression analysis of the myelodysplastic syndrome groups

Unsupervised hierarchical clustering was used to group 123 MDS samples based on their frequencies of all six HSPC subpopulations measured by flow cytometry and normalized to the proportion of the corresponding parent cell compartment (CD34^+^CD38^−^ or CD34^+^CD38^+^). A dendrogram built using Ward’s clustering method^33^ was used to identify distinct MDS groups, and PCA was used to identify the main sources of variation among samples. Frequencies were further used as variables in a logistic regression model to provide a quantitative classifier for the data. The regressed classification equation was log(odds) = 0.5567 × %GMP + 0.4198 × %LMPP − 32.004, where ‘odds’ is the odds that the sample should be assigned to group 2, the GMP-pattern group. The magnitude of the log(odds) value indicates the confidence in the classification (95% of the cases in our analysis were classified with at least 90% confidence). The application of this classifier to the samples is shown in Fig. 1a. Ward’s clustering, PCA and linear correlation analyses were conducted with R statistical software (v3.4.2).

### Flow cytometry and FACS

Quantitative flow cytometric analyses and FACS of human live MNCs and CD34^+^ cells were performed using standard staining protocols for established antigen panels (Supplementary Table 1) and antibodies to CD2 (RPA-2.10; 1:20 dilution), CD3 (SK7; 1:10 dilution), CD14 (MφP9; 1:20 dilution), CD19 (SJ25C1; 1:10 dilution), CD20 (2H7; 1:10 dilution), CD34 (581; 1:20 dilution), CD56 (B159; 1:40 dilution), CD123 (9F5; 1:20 dilution) and CD235a (HIR2; 1:40 dilution; all from BD Biosciences); CD4 (S3.5; 1:20 dilution), CD11b (ICRF44; 1:20 dilution), CD33 (P67.6; 1:20 dilution) and CD90 (5E10; 1:10 dilution; all from Thermo Fisher Scientific); CD7 (6B7; 1:20 dilution) and CD38 (HIT2; 1:20 dilution; all from BioLegend); CD10 (SJ5-1B4; 1:20 dilution; Leinco Technologies); and CD45RA (HI100; 1:10 dilution; Tonbo Biosciences). The flow cytometry and FACS settings are detailed in Supplementary Table 9.

Flow cytometric analysis of HSPC populations in mouse BM suspensions was performed according to HSPC definitions shown in Supplementary Table 10 and following standard protocols, using the biotin-labeled mouse Lineage Cell Depletion Kit (Miltenyi Biotec), fluorochrome-conjugated streptavidin (1:100 dilution; BD Biosciences) and antibodies to CD34 (RAM34; 1:20 dilution), Sca-1 (D7; 1:100 dilution), CD135/Flt3 (A2F10; 1:40 dilution), CD16/CD32 (93; 1:200 dilution; all from Thermo Fisher Scientific), and CD117/c-Kit (2B8; 1:200 dilution; BD Biosciences, BioLegend or Thermo Fisher Scientific, depending on the fluorochrome). In transplantation experiments, donor cells were identified by concurrent staining with anti-CD45.2 (104; 1:20 dilution; BioLegend). Apoptosis was assessed by incubating previously stained cells with Annexin V (1:40 dilution; Thermo Fisher Scientific). For cell cycle analysis, previously stained cells were fixed and permeabilized with IntraPrep Permeabilization Reagent (Beckman Coulter) and subsequently stained with an anti-Ki67 antibody (SolA15; 1:20 dilution; Thermo Fisher Scientific).

To assess PB reconstitution in BM transplant recipient mice, PB specimens were incubated with red blood cell lysis buffer (Sigma-Aldrich) and stained with a cocktail of antibodies to CD45.1 (A20; 1:100 dilution), CD45.2 (104; 1:40 dilution), Gr-1/Ly-6G/6C (RB6-8C5; 1:200 dilution), CD3ε (145-2C11; 1:100 dilution; all from Thermo Fisher Scientific) and B220/CD45R (RA3-6B2; 1:100 dilution; BD Biosciences).

In patient-derived xenografts, human chimerism was analyzed by staining the mouse BM suspensions with antibodies to mouse CD45 (30-F11; 1:20 dilution; BioLegend) and human CD45 (HI30; 1:10 dilution; BD Biosciences).

Samples used for flow cytometry and FACS were acquired with a BD LSR Fortessa or BD Influx Cell Sorter using BD FACSDiva software, v8.01 (all from BD Biosciences), and the cell populations were analyzed using FlowJo software (v10.5.3, FlowJo). All experiments included fluorescence-minus-one and single-stained controls and were performed at MD Anderson’s South Campus Flow Cytometry & Cell Sorting Core Facility.

### *t*-distributed stochastic neighbor embedding analysis of flow cytometry data

Flow cytometry files (vFCS3.0) were analyzed using FlowJo software. A maximum of 5,000 events were downsampled from the live Lin^−^CD34^+^ population for each patient file. All downsampled data files (*n* = 122) were concatenated into a single file to produce a *t*-SNE map. One file could not be pooled owing to parameter incompatibility. The *t*-SNE analysis was calculated with a perplexity of 30 in 1,000 iterations. CMP-pattern and GMP-pattern MDS samples classified using the log(odds) ratio described above were overlaid on the HSPC *t*-SNE map (Extended Data Fig. 1a).

### Mouse models

Mice were maintained under specific-pathogen-free conditions at MD Anderson and housed in a barrier facility at 25 °C under ambient oxygen conditions in a 12-h light/12-h dark cycle under 50% humidity. All animal experiments were performed with the approval of MD Anderson’s Institutional Animal Care and Use Committee. All animal studies used 12- to 16-week-old mice and both males and females unless otherwise indicated.

Heterozygous G0 *Tert*^*ER/+*^ (control) and late-generation homozygous G5 *Tert*^*ER/ER*^ (MDS-like) mice were generated using a standard breeding protocol of successive generations. The conditional deletion of *Tet2* in the hematopoietic compartment was accomplished by crossing *Vav-Cre* mice^34^ with *Tet2*^*L/L*^ mice^35^ (both from The Jackson Laboratory) to generate *Vav-Cre/Tet2*^*L/L*^ mice. Mice carrying the *Srsf2*^*P95H*^ mutation were obtained by crossing *Mx1-Cre* mice^36^ with *Srsf2*^*P95H*^ mice^37^ (both from The Jackson Laboratory). We further crossed *Mx1-Cre/Srsf2*^*P95H*^ mice with *Runx1*^*L/+*^ mice^38^ (The Jackson Laboratory) to obtain *Runx1*^*L/+*^*/Srsf2*^*P95H*^ mice. Polyinosine-polycytosine (Sigma-Aldrich) was intraperitoneally administered to the transplant recipient mice every other day for five doses. *U2AF1*^*S34F*^*/rtTA* mice^39^, which carried a *U2AF1*^S34F^ mutation inducible via a reverse tetracycline-controlled transactivator (*rtTA*), were obtained from The Jackson Laboratory. *U2AF1*^*WT*^*/rtTA* mice were a generous gift of M. Walter (Washington University in St. Louis). Doxycycline was administered to transplant recipient mice via doxycycline-containing rodent chow (TestDiet).

C57BL/6J (B6) mice and NSG-SGM3 (NSGS) mice were obtained from The Jackson Laboratory. Only female recipient NSGS mice were used to develop patient-derived xenografts.

### Competitive bone marrow transplantation experiments

Recipient CD45.1^+^ B6 mice were lethally irradiated with a total of 10.6 Gy delivered in two doses 2 h apart and then injected via tail vein with a single-cell suspension of 0.5–1 × 10^6^ CD45.2^+^ donor and CD45.1^+^ competitor BM cells. Donor-derived PB reconstitution was assessed 6 weeks after transplantation, and mice with similar mean chimerism were randomized to treatment groups.

In human xenograft experiments, recipient NSGS mice were sublethally irradiated with a single dose of 2.6 Gy and then injected via tail vein with a single-cell suspension containing 0.5 × 10^6^ MDS-L cells or 0.2–1 × 10^6^ primary T cell-depleted BM cells isolated from MDS patients whose disease progressed to sAML. Engraftment was assessed every 2 weeks as described above. When human cells were first detected above a minimum confidence level (≥1%), mice with similar mean chimerism were randomized and treatment was initiated (usually, 4–6 weeks after transplantation). After 2–3 weeks of therapy, mean human chimerism in vehicle-treated mouse groups was as high as 10–90% in every independent patient-derived xenograft experiment.

### In vivo treatment follow-up and endpoint analyses

During treatment, PB samples were periodically collected in EDTA-coated tubes, and complete blood counts were performed with an automated ABX Pentra Hematology Analyzer (Horiba).

At the end of treatment, the mice were killed and autopsied, and their rear legs and, occasionally, spleens were resected for analysis. For BM biopsies, tibias and spleens were fixed in 10% neutral-buffered formalin (Sigma-Aldrich) overnight, decalcified in Cal-Ex (tibias only; Thermo Fisher Scientific) for 24 h, and then transferred into 70% ethanol and stored at room temperature (RT) for a minimum of 24 h for dehydration. Fixed tissues were embedded in paraffin according to standard protocols. For BM flow cytometric analyses, femurs and tibias, or spleens, were crushed in the presence of a 2% FBS/PBS solution, and the cell suspensions were passed through 30-µm pre-separation filters (Miltenyi Biotec). Red blood cells were eliminated from splenic samples using BD Lysing Buffer (BD Biosciences). Nucleated cells were then counted to assess total cellularity and analyzed by flow cytometry. Absolute cell numbers were calculated based on BM or spleen cellularity and frequency of specific cell populations. In experiments using non-transplanted mice, the BM cellularity was normalized to the weight of individual animals to account for differences in body size.

### Drugs and treatments

5-aza (Sigma-Aldrich) was reconstituted in PBS. Cultured cells were treated with 0.5 μM 5-aza. Mice were intraperitoneally injected with 5-aza at 2.5 mg per kilogram body weight per day for 1 week per treatment cycle unless otherwise indicated.

ABT-199 was provided by AbbVie. For in vitro experiments, ABT-199 was dissolved in DMSO and diluted in PBS. Cells were treated with 50 nM ABT-199 unless otherwise indicated. For in vivo experiments, fresh suspensions of ABT-199 in a mix of Phosal 50 PG (Lipoid), polyethylene glycol (PEG) 400 and ethanol (60:30:10) were prepared weekly and stored at 4 °C. ABT-199 was administered by gavage at a dose of 100 mg per kilogram body weight per day.

BMS-345541 was purchased from Selleck Chemicals. For in vitro experiments, BMS-345541 was reconstituted in DMSO and diluted in sterile PBS. Cells were treated with 5 μM BMS-345541. For in vivo experiments, suspensions of BMS-345541 in a mix of DMSO, PEG 300, Tween 80 and water (5:30:5:60) were freshly prepared and stored at RT. BMS-345541 was administered every other day by gavage at 75 mg per kilogram body weight per day for 3 weeks (ten doses total).

Human-recombinant TNF (R&D Systems) was reconstituted in 0.1% FBS/sterile PBS. Cells were treated with 2.5 ng ml^−1^ TNF.

### Histological analyses

Formalin‐fixed paraffin‐embedded mouse BM sections (3 μm) were prepared for antibody detection and H&E staining according to standard procedures. Chromogenic IHC was performed at MD Anderson’s Research Histology Core Laboratory using an anti-cleaved caspase-3 antibody (Biocare Medical; 1:100 dilution) or an anti-human CD45 antibody (D9M8I; Cell Signaling Technology; 1:200 dilution) and hematoxylin counterstaining. Microscopy preparations were analyzed by a dedicated hematopathologist at MD Anderson.

All human BM biopsy specimens were routinely collected and processed in MD Anderson’s Department of Hematopathology. Specimens were fixed in 10% neutral-buffered formaldehyde, and core biopsy specimens were further decalcified using 10% formic acid for 3 h at 50 °C in a microwave processor. Specimens were embedded in paraffin, and 4-μm sections were prepared for antibody detection. IHC was performed at the Dana-Farber/Harvard Cancer Center Specialized Histopathology Core. Formalin‐fixed paraffin‐embedded samples were stained with anti-human BCL-2 (124; 1:500 dilution; Dako, Agilent), and anti-human phospho-p65 (phospho-S536; 1:750 dilution; Abcam) and counterstained with hematoxylin.

### Whole-exome sequencing

Total DNA from BM MNCs, neutrophils and T cells was extracted using the DNeasy Blood & Tissue Kit and AllPrep DNA/RNA Micro Kit (Qiagen), respectively. DNA from sorted HSC populations (25–2,000 cells per sample) was extracted and amplified using the REPLI-g Cell WGA & WTA Kit (Qiagen). Quality checks and quantification were performed with a Qubit Fluorometer (Thermo Fisher Scientific) and a Fragment Analyzer (Advanced Analytical Technologies). Library preparation was performed at MD Anderson’s Advanced Technology Genomics Core Facility using the SureSelect XT Automated Library Prep Kit (Agilent) directly in samples with more than 200 ng of DNA. For samples with 50–200 ng of DNA, the SureSelect XT Low Input Library Prep Kit (Agilent) was used. All samples were sequenced with a HiSeq 4000 System (Illumina) generating 150-nucleotide paired-end reads. Raw data analysis was performed at the McDonnell Genome Institute at Washington University in St. Louis and the Department of Genomic Medicine at MD Anderson.

At Washington University in St. Louis, data were imported and aligned to the human reference (GRCh38.d1.vd1+ chr21:6427259-6580181_mask) using the Burrows–Wheeler Aligner (BWA)-MEM alignment algorithm (http://sourceforge.net/projects/bio-bwa/files/). Duplicate reads were tagged using Picard MarkDuplicates (GATK, Broad Institute). Somatic mutations were called using the genome/analysis-workflow somatic exome processing pipeline (v1.4-beta.1). Somatic variants were annotated using the Ensembl Variant Effect Predictor (release 93.2). The entire pipeline is available on GitHub (10.5281/zenodo.2580356). Only high-confidence variants (those reported by at least two different variant-calling tools to have a VAF ≥ 5%) were considered.

At MD Anderson, raw sequencing data were converted to a fastq format and aligned to the human genome (hg19, GRCh37) using the BWA-MEM algorithm. The aligned BAM files were processed using Picard and GATK with default parameters and then variants were called using MuTect and Pindel against pooled unmatched normal sequences developed in-house. Variants with low-quality sequencing, no obvious protein-coding change or common polymorphisms with a population frequency of 0.14% in public variant databases were filtered out.

All somatic mutation candidates were manually filtered using the 100-gene FLAGS list of frequent false positives in exome-captured human genes^40^, a curated list of 1,056 genes generated at Washington University in St. Louis, based on relevant publications^41–50^ and a previously established list of 81 known leukemia-mutated genes used for targeted amplicon-based next-generation sequencing at MD Anderson^51,52^. Noncoding variants in splicing regions were only considered when they were confirmed to be pathogenic.

We inferred the clonal composition and mutational hierarchies using PyClone (v0.13.1)^53^. The mutations belonging to the dominant clones with the highest cellular prevalence were deemed to be founder mutations. Co-mutation patterns were plotted using Circos^54^.

For tracking variants in HSC populations, a confidence interval of 95% was used to establish a minimum reference read number cutoff of 60 for a given variant to be considered negative (that is, having a VAF <5%) in a sample. Cases with no variant reads or with fewer than 60 reference reads were reanalyzed by targeted sequencing.

For comparing the number of mutations and acquisition rate in HSCs, we used a 5% VAF cutoff and considered only patients with reads in at least two cell populations. The Wilcoxon rank-sum test was used to compare the groups, and the Wilcoxon signed-rank test was used to compare cell populations within a single group. The difference in mutation gain rates between groups was estimated using a generalized linear mixed effects model in which LT-HSCs, MPPs and LMPPs were equidistant from one another (that is, 1-2-3). The model included the cell type, patient group and the interaction term (cell type × group) as fixed effects and the patient as the random effect.

### Targeted gene sequencing

Selected samples were re-sequenced at MD Anderson’s Advanced Technology Genomics Core facility. We used a previously established custom SureSelect panel of 295 genes (Agilent) that are recurrently mutated in hematological malignancies^55^. DNA from HSC populations was fragmented and bait-captured in solution according to the manufacturer’s protocols. Captured DNA libraries were then sequenced using a NovaSeq sequencer (Illumina) with 76-bp paired-end reads. Raw sequencing data were analyzed using the MD Anderson Department of Genomic Medicine pipeline explained above.

### scRNA-seq

For scRNA-seq, 3,000 Lin^−^CD34^+^ cells were sorted by FACS. Sample preparation and sequencing were performed at MD Anderson’s Advanced Technology Genomics Core facility. Sample concentration and cell suspension viability were evaluated using a Countess II FL Automated Cell Counter (Thermo Fisher Scientific) and manual counting. Samples were normalized for input onto the Chromium Single Cell A Chip Kit (10x Genomics) for reverse transcription. The pooled single-stranded, barcoded cDNA was amplified and fragmented for library preparation with appropriate sequence primer sites and adaptors added for sequencing on a NextSeq 500 sequencer (Illumina).

After sequencing, fastq files were generated using the cellranger mkfastq pipeline (v3.0.2). The raw reads were mapped to the human reference genome (refdata-cellranger-GRCh38-3.0.0) using the cellranger count pipeline. Multiple samples were aggregated using the cellranger aggr pipeline. The digital expression matrix was analyzed with the R package Seurat (v3.0.2)^56^. Cells with fewer than 500 unique molecular identifiers or greater than 50% mitochondrial expression were removed from further analysis. PCA, *t*-SNE and UMAP were used to reduce the dimensions of the data, and the first two dimensions were used in the plots. Cell types were annotated based on the marker genes and their match to canonical markers.

The R package Monocle (v2.10.1)^57^ was used to perform the pseudotime analyses. Expressed genes were defined as those expressed in more than five cells or that had a mean expression level greater than 0.05.

### Total RNA sequencing

Total RNA from sorted HSC populations (≥250 cells) was purified using the PicoPure RNA Isolation Kit (Thermo Fisher Scientific). Quality checks and quantification were performed with the 2100 Expert Bioanalyzer (Agilent). cDNA libraries were prepared at MD Anderson’s Advanced Technology Genomics Core facility using the Ovation Single Cell RNA-Seq System (NuGEN Technologies) according to manufacturer’s specifications. RNA-seq was performed with the HiSeq 4000 System (Illumina) using the standard paired-end protocol. An average of 60 million 76-bp reads were generated per sample.

The mapping of the RNA-seq reads to the human reference genome hg19 was performed with Tophat2 (John Hopkins University Center for Computational Biology). The quality of the reads was verified using FASTQC software (v0.11.8, Babraham Institute). HTseq software^58^ (v0.11.2) was used to summarize the gene expression counts from the Tophat2 alignment data after sorting the BAM files. Unsupervised analysis was performed using PCA and hierarchical clustering to verify any outliers and the overall similarities or differences among all samples. Differential expression analysis was performed on contrasts of interest using the DEseq2 package^59^.

Enriched pathways were identified using GSEA (http://www.broadinstitute.org/gsea/msigdb/annotate.jsp) and Metascape (https://metascape.org/)^60^.

### Colony-formation assays

Human CMPs, GMPs or MEPs were sorted directly into sterile StemSpan Serum-Free Expansion Medium (STEMCELL Technologies). Cell suspensions were diluted in human cytokine-supplemented MethoCult methylcellulose medium (STEMCELL Technologies) at a 1:10 ratio, plated in two 35 mm × 10 mm culture dishes (~500–2,000 cells per dish), and incubated at 37 °C in a 5% CO_2_ atmosphere. After 14 d, the colonies were scored under a phase microscope; standard benzidine staining was used to identify erythroid colonies.

### Primary cell culture assays

FACS-purified human CD34^+^ HSPCs were sorted directly into cytokine-free sterile RPMI medium supplemented with 10% FBS, 1% penicillin–streptomycin and 0.1% amphotericin B and plated in 48-well plates previously seeded with low-passage (P ≤4) healthy BM-derived human MSCs. Co-cultures were incubated at 37 °C in a 5% CO_2_ atmosphere. After treatment, cells were harvested and stained for quantitative flow cytometric analysis using the antigen panel detailed in Supplementary Table 1, with AccuCheck Counting Beads (Thermo Fisher Scientific) added to each tube.

In live microscopy assays, FACS-purified Lin^−^CD34^+^ cells were sorted into PBS and stained with 0.5 µM IncuCyte CytoLight Rapid Red Reagent (Essen Biosciences). The cells were then washed and plated in 96-well plates pre-seeded with MSCs. After 12–16 h in culture, the indicated drugs were added to each well in the presence of Caspase-3/7 Green Apoptosis Assay Reagent (1:1,000 dilution; Essen Biosciences). Co-cultures were incubated in an IncuCyte S3 Live Cell Analysis System (Essen Biosciences) placed in an incubator at 37 °C and 5% CO_2_. Live microscopy images of the co-cultures were captured every hour using the IncuCyte S3 software (v2017A, Essen Biosciences) at ×20 magnification.

### Mesoscale discovery assays

MDS-L cells isolated from the BM of xenografts treated with ABT-199 were resuspended in Meso Scale Discovery (MSD) Tris Lysis Buffer (MSD) with protease inhibitor for 10 min. Protein samples (10 µg) were subsequently added to streptavidin-coated 96-well plates pre-immobilized with antibodies to BCL-2, BCL-XL and MCL-1 (MSD 96, 4-Spot Abbvie BCL-2 3-Plex SECTOR plate, MSD) and incubated for 1 h at RT. The protein samples were washed three times with MSD Wash Buffer, and then 1 µg ml^−1^ SULFO-TAG anti-human BIM rabbit monoclonal antibody or the combination of 1 µg ml^−1^ SULFO-TAG anti-human BCL-2, 0.5 µg ml^−1^ SULFO-TAG anti-human BCL-XL, and 0.5 µg ml^−1^ SULFO-TAG anti-human MCL-1 rabbit monoclonal antibodies (MSD) was added to the protein samples, which were incubated for 1 h at RT with rotation at 40*g* and then washed an additional three times. Finally, 150 µl of MSD Read Buffer T was added per well, and fluorescence was measured with an MSD Sector Imager 6000.

### Western blots

Cell pellets were resuspended in Mammalian Cell & Tissue Extraction Kit buffer (BioVision Incorporated) and incubated for 15 min with gentle shaking. Lysates were then collected after centrifugation at 13,500*g* and 4 °C for 20 min. The amount of protein was quantified using the Qubit Protein Assay Kit and a Qubit Fluorometer (Thermo Fisher Scientific). SDS–PAGE and western blotting were performed following standard protocols. Blotted membranes were incubated with primary monoclonal antibodies to human BCL-2 (124; 1:1,000 dilution; Dako), p65 (D14E12; 1:1,000 dilution; Cell Signaling Technology), phospho-p65 (Ser536, 93H1; 1:1,000 dilution; Cell Signaling Technology) and vinculin (hVIN-1; 1:2,000 dilution; Sigma-Aldrich) and with secondary digital anti-mouse and anti-rabbit antibodies (1:2,000 dilution; Kindle Biosciences). Membranes were developed using the SuperSignal West Pico PLUS Chemiluminescent Substrate (Thermo Fisher Scientific) in a KwikQuant Imager (Kindle Biosciences). Vinculin was used as a loading control, and lysates from HL-60 and JJN3 cells were used as positive controls.

### Cytometry by time-of-flight analysis

Sequential samples were stained with a 52-antibody panel (Supplementary Table 11). To minimize variability, we used the Cell-ID 20-Plex Pd barcoding Barcoding kit (Fluidigm). Cytometry by time-of-flight (CyTOF) data were processed and analyzed based on the standard pipeline. Data were normalized by the R/Bioconductor package CATALYST (v1.18.0). Cell populations were identified by unsupervised clustering using FlowSOM^61^ (v2.2) and ConsensusClusterPlus^62^ (v1.5.8) packages. Cells were assigned to the 100 grid points based on their similarities. A minimal spanning tree was established for graphical representation, and metacluster was performed with consensus clustering. Clusters were merged to major lineages, and cell populations were identified based on conventional cell-type-specific markers. Clustering information was visualized using *t*-SNE by CytoTREE^63^ (v1.4).

### Statistical analysis and figures

Experimental data were analyzed with Prism 8 software (GraphPad). Figure legends indicate the statistical tests used in each experiment. Non-Gaussian distributions were detected using the D’Agostino–Pearson normality test. Mouse data were analyzed by pooling biological replicates from different experiments. In analyses involving human samples, investigators were blinded to sample annotations and patient outcomes. Experimental design diagrams were made using BioRender.com.

### Reporting Summary

Further information on research design is available in the Nature Research Reporting Summary linked to this article.

## Online content

Any methods, additional references, Nature Research reporting summaries, source data, extended data, supplementary information, acknowledgements, peer review information; details of author contributions and competing interests; and statements of data and code availability are available at 10.1038/s41591-022-01696-4.

## Supplementary information

Supplementary Information Supplementary Figs. 1–11

Reporting Summary

Supplementary Tables 1–11

## Data Availability

Datasets generated in this study by RNA-seq in HSCs and progenitors and scRNA-seq are accessible at GSE178840 and GSE137429, respectively. All can be accessed from the super-series GSE136816. Data generated by DNA-seq are accessible at the European Genome-phenome Archive under accessions S00001003867, S00001003869 and S00001003868 and at BioProject under PRJNA737460. Source data are provided with this paper.

## Source data

Source Data Fig. 1 Statistical source data.
Source Data Fig. 2 Statistical source data.
Source Data Fig. 3 Statistical source data.
Source Data Fig. 4 Statistical source data.
Source Data Extended Data Fig. 1 Statistical source data.
Source Data Extended Data Fig. 2 Statistical source data.
Source Data Extended Data Fig. 3 Statistical source data.
Source Data Extended Data Fig. 4 Statistical source data.
Source Data Extended Data Fig. 5 Statistical source data.
Source Data Extended Data Fig. 6 Statistical source data.
Source Data Extended Data Fig. 7 Statistical source data.
Source Data Extended Data Fig. 8 Statistical source data.
Source Data Extended Data Fig. 9 Statistical source data.
Source Data Extended Data Fig. 9 Unprocessed western blots.
Source Data Extended Data Fig. 10 Statistical source data.

## Code availability

The R code used for the clustering and classification of MDS sample flow cytometry data can be obtained from the authors upon request by email.
