# Author Correction: Stem cell architecture drives myelodysplastic syndrome progression and predicts response to venetoclax-based therapy

**DOI:** 10.1038/s41591-022-01827-x

**Published:** 2022-04-28

**Authors:** Irene Ganan-Gomez, Hui Yang, Feiyang Ma, Guillermo Montalban-Bravo, Natthakan Thongon, Valentina Marchica, Guillaume Richard-Carpentier, Kelly Chien, Ganiraju Manyam, Feng Wang, Ana Alfonso, Shuaitong Chen, Caleb Class, Rashmi Kanagal-Shamanna, Justin P. Ingram, Yamini Ogoti, Ashley Rose, Sanam Loghavi, Pamela Lockyer, Benedetta Cambo, Muharrem Muftuoglu, Sarah Schneider, Vera Adema, Michael McLellan, John Garza, Matteo Marchesini, Nicola Giuliani, Matteo Pellegrini, Jing Wang, Jason Walker, Ziyi Li, Koichi Takahashi, Joel D. Leverson, Carlos Bueso-Ramos, Michael Andreeff, Karen Clise-Dwyer, Guillermo Garcia-Manero, Simona Colla

**Affiliations:** 1 Department of Leukemia, The University of Texas MD Anderson Cancer Center, Houston, TX USA; 2 Molecular Biology Institute, University of California, Los Angeles, Los Angeles, CA USA; 3 Division of Rheumatology, Department of Internal Medicine, Michigan Medicine, University of Michigan, Ann Arbor, MI USA; 4 Department of Medicine and Surgery, University of Parma, Parma, Italy; 5 Department of Bioinformatics and Computational Biology, The University of Texas MD Anderson Cancer Center, Houston, TX USA; 6 Department of Genomic Medicine, The University of Texas MD Anderson Cancer Center, Houston, TX USA; 7 Department of Biostatistics, The University of Texas MD Anderson Cancer Center, Houston, TX USA; 8 Department of Hematopathology, The University of Texas MD Anderson Cancer Center, Houston, TX USA; 9 AbbVie Oncology Discovery, Chicago, IL USA; 10 Department of Stem Cell Transplantation and Cellular Therapy, The University of Texas MD Anderson Cancer Center, Houston, TX USA; 11 McDonnell Genome Institute, Washington University in St. Louis, St. Louis, MO USA; 12 Istituto Romagnolo per lo Studio dei Tumori ‘Dino Amadori’, Meldola, Italy

**Keywords:** Stem cells, Cancer stem cells

Correction to: *Nature Medicine* 10.1038/s41591-022-01696-4, published online 3 March 2022.

In the version of this article initially published, the name of the Edward P. Evans Foundation was shown incorrectly in the first sentence of the Acknowledgements. The name has been corrected in the HTML and PDF versions of the paper.

