# Supplementary material for: Stem cell architecture drives myelodysplastic syndrome progression and predicts response to venetoclax-based therapy
Source: Nat Med. 2022 Mar 3;28(3):557–67. doi: 10.1038/s41591-022-01696-4 (PMC8938266; doi:10.1038/s41591-022-01696-4)
Supplement: Supplementary file 1 — Supplementary Figs. 1–11 [file 41591_2022_1696_MOESM1_ESM.pdf]

nature portfolio

https://doi.org/10.1038/s41591-022-01696-4

Supplementary information

Stem cell architecture drives myelodysplastic syndrome progression and predicts response to venetoclax-based therapy

In the format provided by the authors and unedited

Supplementary Figure 1. MDS have two abnormal BM HSPC architectures.

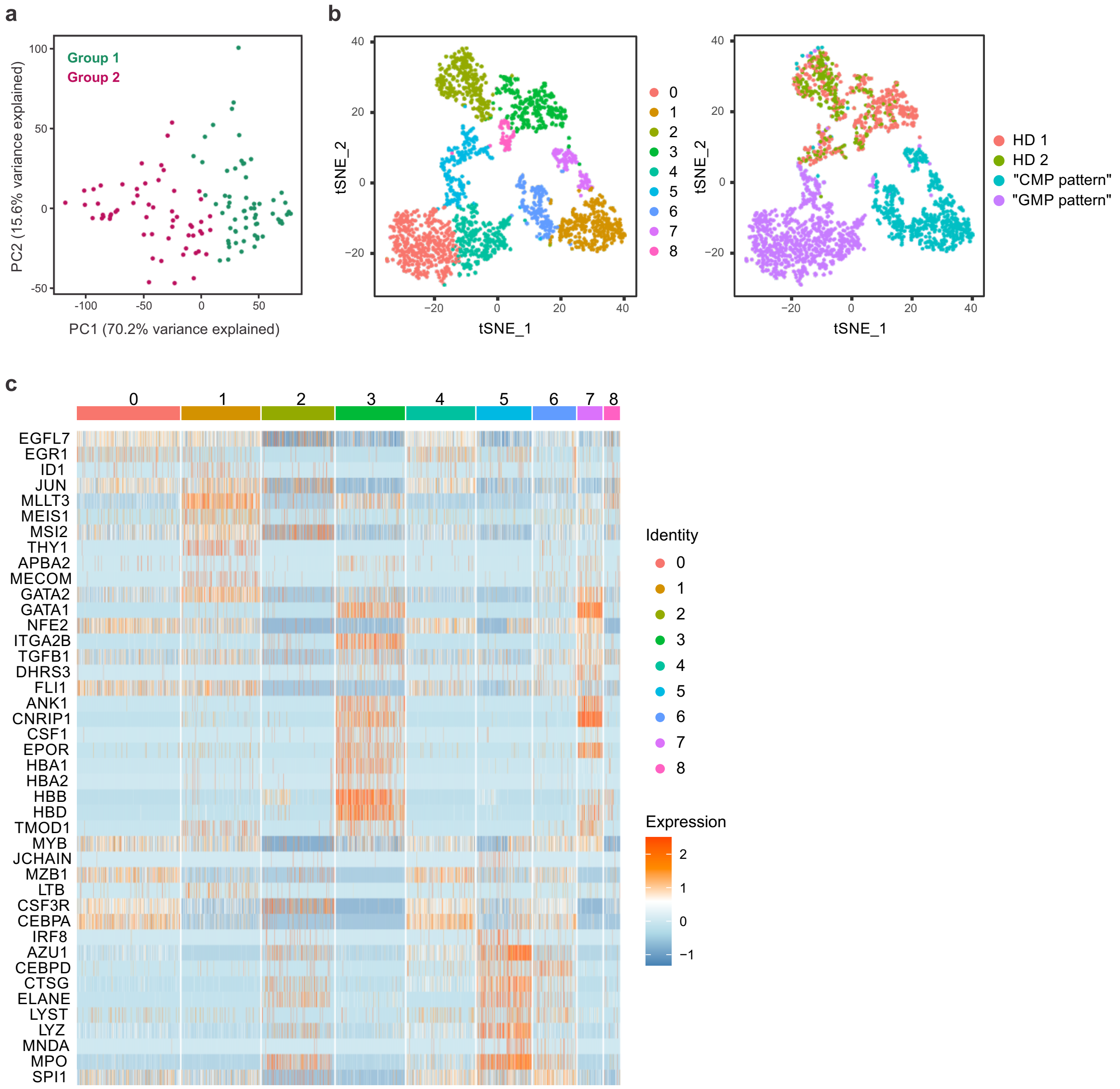

(a) PCA of the frequencies of immunophenotypic HSPC subpopulations from 123 MDS samples. Two groups (group 1, n=64; group 2, n=59) were identified by unsupervised hierarchical clustering. Each symbol represents one sample. **(b)** tSNE maps of scRNA-seq data displaying 2,550 Lin$^-$CD34$^+$ cells isolated from the BM of two representative HD samples, a representative "CMP pattern" MDS sample, and a representative "GMP pattern" MDS sample. Each symbol represents one cell. Different colors indicate the differential gene expression cluster (left) and sample origin (right) of each cell. **(c)** Heatmap showing the differential expression of a curated set of stem cell and lineage-specific genes in the HD and MDS Lin$^-$CD34$^+$ cells clusters shown in (b).

Supplementary Figure 2. MDS HSPC architectures have myeloid-biased progenitors.

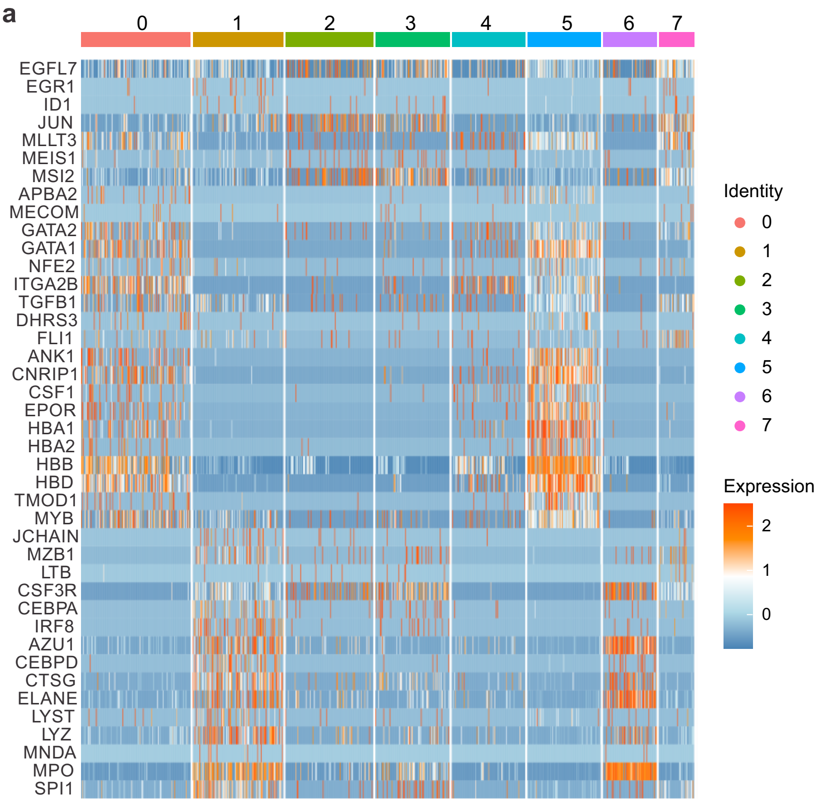

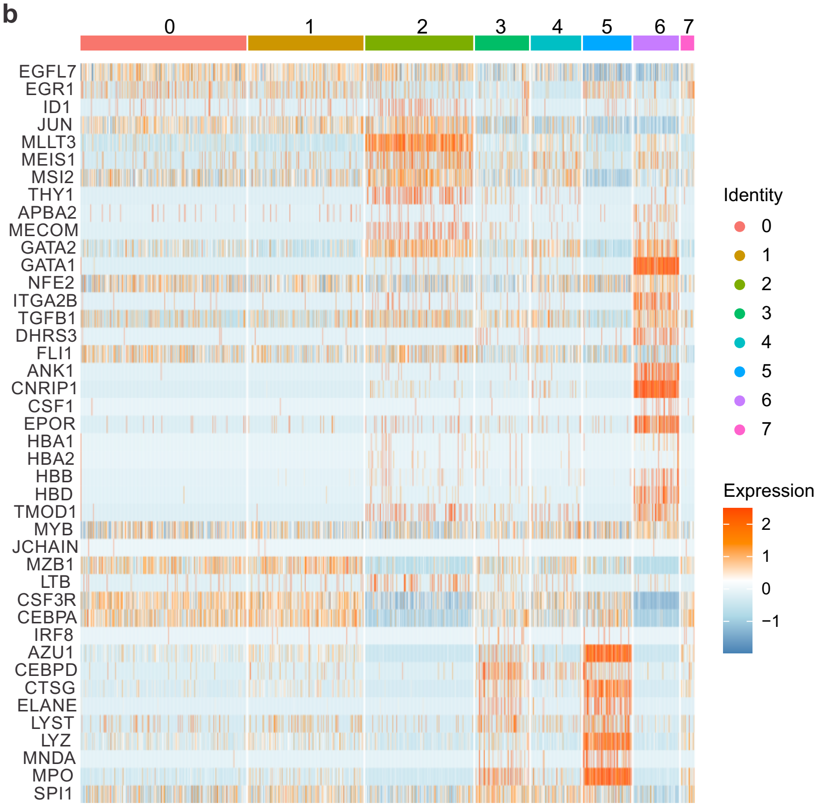

(a) Differential expression of a curated set of stem cell and lineage-specific genes in the HD Lin$^-$CD34$^+$ cells shown in Extended Data Figure 3a. **(b)** Differential expression of a curated set of stem cell and lineage-specific genes in the MDS Lin$^-$CD34$^+$ cells shown in Fig. 1e.

Supplementary Figure 3. Founder mutations occur in LT-HSCs in most “CMP pattern” MDS.

1-142	LT-HSCs	MPPs	LMPPs	MNCs
BCOR	0.40	0.96	0.93	0.87
U2AF1	0.48	0.96	0.89	0.67
RUNX1	0.24	0.43	0.78	0.55

1-193	LT-HSCs	MPPs	LMPPs	MNCs
U2AF1	1.00	X	X	0.26
ASXL1	0.89	1.00	X	0.25
ZRSR2	0	X	X	0.10
ARID5B	0	0	X	0.06

1-237	LT-HSCs	MPPs	LMPPs	MNCs
IL6ST	0.46	0.46	0.58	0.06
DDX41	0.56	0.21	0.70	0.04

1-242	LT-HSCs	MPPs	LMPPs	MNCs
ASXL1	X	0.26	X	0.35
TET2	0.56	0	X	0.22
JAK2	0	0	0	0.14

1-271	LT-HSCs	MPPs	LMPPs	MNCs
SF3B1	0.90	0.41	0.23	0.44

1-311	LT-HSCs	MPPs	LMPPs	MNCs
TP53 p.G266fs	X	1.00	0.73	0.38
TP53 p.Y234C	0.50	0.82	0.07	0.38
SF3B1	0	0	0	0.06

17-02	LT-HSCs	MPPs	LMPPs	MNCs
TET2 p.R1878P	0.05	0	X	0.40
PMEPA1	0.40	0.40	X	0.38
ASXL1	0.50	0.93	X	0.31
TET2 p.G1172GX	0.17	0.01	X	0.30

17-20	LT-HSCs	MPPs	LMPPs	MNCs
TET2	0	0.52	0.73	0.50
SF3B1	0	0.12	0	0.42
ARID1B	X	0.08	X	0.16

17-29	LT-HSCs	MPPs	LMPPs	MNCs
SF3B1	0.17	0.12	0.50	0.32

18-07	LT-HSCs	MPPs	LMPPs	MNCs
SF3B1	1.00	0.11	0.99	0.48
ASXL1	X	X	X	0.11
TET2	X	X	0	0.11

18-10	LT-HSCs	MPPs	LMPPs	MNCs
TET2 p.R1404*	0.23	0.90	X	0.46
SF3B1	0.39	0.21	X	0.39
TET2 p.I1228F	0	0	X	0.06

C2-150	LT-HSCs	MPPs	LMPPs	MNCs
U2AF1	0.54	0.44	0.37	0.43
ASXL1	0.34	0	0	0.39
ZC3H11A	0	0	0	0.06

C2-29	LT-HSCs	MPPs	LMPPs	MNCs
SF3B1	0.35	0.46	0.19	0.40
PIK3CD	0.46	X	0.67	0.33
PLXNB2	X	X	X	0.10
PPM1D	0	0.07	0	0.07

C2-37	LT-HSCs	MPPs	LMPPs	MNCs
TP53	0.57	0.98	0.37	0.20
TYR	0.45	0.56	0.36	0.19
EZH2	0.39	0.38	0.22	0.18
CIITA	0.22	0.44	0.18	0.17

C2-66	LT-HSCs	MPPs	LMPPs	MNCs
SF3B1	0.41	0.56	0.36	0.45

C2-73	LT-HSCs	MPPs	LMPPs	MNCs
TET2 p.H1902R	0.26	0.64	0.02	0.43
TET2 p.R1537X	0.58	0.16	X	0.42
ASXL1	0.24	0.34	0.14	0.37

C2-90	LT-HSCs	MPPs	LMPPs	MNCs
TET2	0.99	0.97	0.94	0.92
NF1	0.58	0.57	0.74	0.44
SRSF2	0	1.00	1.00	0.41

C2-98	LT-HSCs	MPPs	LMPPs	MNCs
ASXL1	0.070	0.004	0.173	0.414
U2AF1	0.096	0.088	0.483	0.381
SETBP1	0.209	0	0	0.105
CREBBP	0	0	0	0.051

C2-100	LT-HSCs	MPPs	LMPPs	MNCs
SF3B1	0.82	0.67	0.26	0.31
TET2	0	0.50	0	0.21

C2-102	LT-HSCs	MPPs	LMPPs	MNCs
IDH2	0.10	0.70	0.34	0.39
SRSF2	1.00	0.53	1.00	0.36
TP53	0.10	0.46	0.35	0.31
JAK2	0.99	0.21	0.54	0.23
ASXL1	0.80	0.08	0.01	0.14
EP300	0.07	0.35	0	0.14
STAT5B	0	0	0	0.08
RUNX1	0.07	0.13	0.01	0.07
SLTM	0.07	0.06	0.09	0.06

C2-105	LT-HSCs	MPPs	LMPPs	MNCs
ASXL1	0.53	0.15	0.23	0.41
SRSF2	X	X	X	0.28
EZH2	X	0.05	0.07	0.26
RUNX1	0	0.99	0.06	0.26
ETNK1	0.13	0.16	0.12	0.23
STAG2	0	0.21	0.01	0.10

C2-106	LT-HSCs	MPPs	LMPPs	MNCs
SMC3	0.09	0.09	0.33	0.12

C2-112	LT-HSCs	MPPs	LMPPs	MNCs
STAG2	0.845	0.874	0.439	0.838
EZH2	0.069	0.740	0.657	0.809
ASXL1	0.233	0.025	0	0.289
PHF6	0	0	0	0.054

VAFs of somatic mutations in oncogenes and leukemia-relevant genes in HSC populations and total BM MNCs from 23 baseline “CMP pattern” MDS samples. VAFs were not corrected for copy-number variation. Crossed-out boxes indicate missing cell populations or insufficient reads in a specific locus to confidently claim the absence of the corresponding mutation in that population.

Supplementary Figure 4. Founder mutations occur in LT-HSCs in most “GMP pattern” MDS.

1-151

	LT-HSCs	MPPs	LMPPs	MNCs
STAG2	0.81	0.80		0.33
NTN4	0	0.13	1.00	0.29
SF3B1	0.21	0.34	0.60	0.28

1-194

	LT-HSCs	MPPs	LMPPs	MNCs
SF3B1	0.63	0.65	0.34	0.44

1-225

	LT-HSCs	MPPs	LMPPs	MNCs
ASXL1	0.90	0.44	0.29	0.10

1-248

	LT-HSCs	MPPs	LMPPs	MNCs
IDH1	0	0.91	0.85	0.32
RUNX1	0	0		0.29

1-274

	LT-HSCs	MPPs	LMPPs	MNCs
ASXL1 p.H630fs	0		0.19	0.17
ASXL1 p.E727X	0.09		0	0.07
ASXL1 p.G642fs	0.13	0.73	0.42	0.04

17-33

	LT-HSCs	MPPs	LMPPs	MNCs
EZH2 p.F670L	0.67		1.00	0.44
ASXL1			0	0.42
SF3B1	0.89	0	0.25	0.38
EZH2 p.V742A	0.27	0.50	0.05	0.36
RUNX1				0.35
IDH1	0.09	0	1.00	0.33

18-05

	LT-HSCs	MPPs	LMPPs	MNCs
TET2		0.85	0.74	0.47
ASXL3		0		0.46
PHF6	0.43	0.94		0.43
TET2		0.85	0.74	0.41

C2-33

	LT-HSCs	MPPs	LMPPs	MNCs
STAG2	0.39	0.06	1.00	0.50
ETNK1	0.63	0.30	0.48	0.42
ASXL1	0.21	0.19	0.15	0.22
RAD21	0.06	0.07	0	0.12
RUNX1 p.P425fs	0.11	0.07		0.11
RUNX1 p.F330_S331delins	0.11	0.06	0.17	0.08
KIT	0.13	0	0	0.07

C2-53

	LT-HSCs	MPPs	LMPPs	MNCs
WT1	0	0	0.56	0.47
ZNF814	0.38	0.48	0.14	0.19

C2-89

	LT-HSCs	MPPs	LMPPs	MNCs
DNMT3A	0	0.36	0.42	0.15
ALK	0.04	0.31	0.43	0.14
TET2 p.R1537X	0.43	0.20	0.11	0.13
TET2 p.E1454fs	0	0.06	0.29	0.11

C2-92

	LT-HSCs	MPPs	LMPPs	MNCs
SF3B1	0.71	0.15	0.64	0.37

C2-101

	LT-HSCs	MPPs	LMPPs	MNCs
RUNX1		0.06	0.36	0.33
PTPN11	0	0.12	0.13	0.27

C2-110

	LT-HSCs	MPPs	LMPPs	MNCs
SF3B1	0.04	0.60	0.67	0.50
RUNX1		0.12	0.27	0.30
ASXL1	1.00	0.16	0.18	0.23
RARA	1.00	0.08	0.11	0.14
SMC3	1.00	0.27	0.10	0.08
STAG2	0.22	0.01	0.01	0.08
TET2	0	0	0	0.08
REV3L	0.02	0.02	0.02	0.05

VAFs of somatic mutations in oncogenes and leukemia-relevant genes in HSC populations and total BM MNCs from 13 baseline “GMP pattern” MDS samples. VAFs were not corrected for copy-number variation. Crossed-out boxes indicate missing cell populations or insufficient reads in a specific locus to confidently claim the absence of the corresponding mutation in that population.

Supplementary Figure 5. HMA therapy does not correct abnormal MDS HSPC architectures.

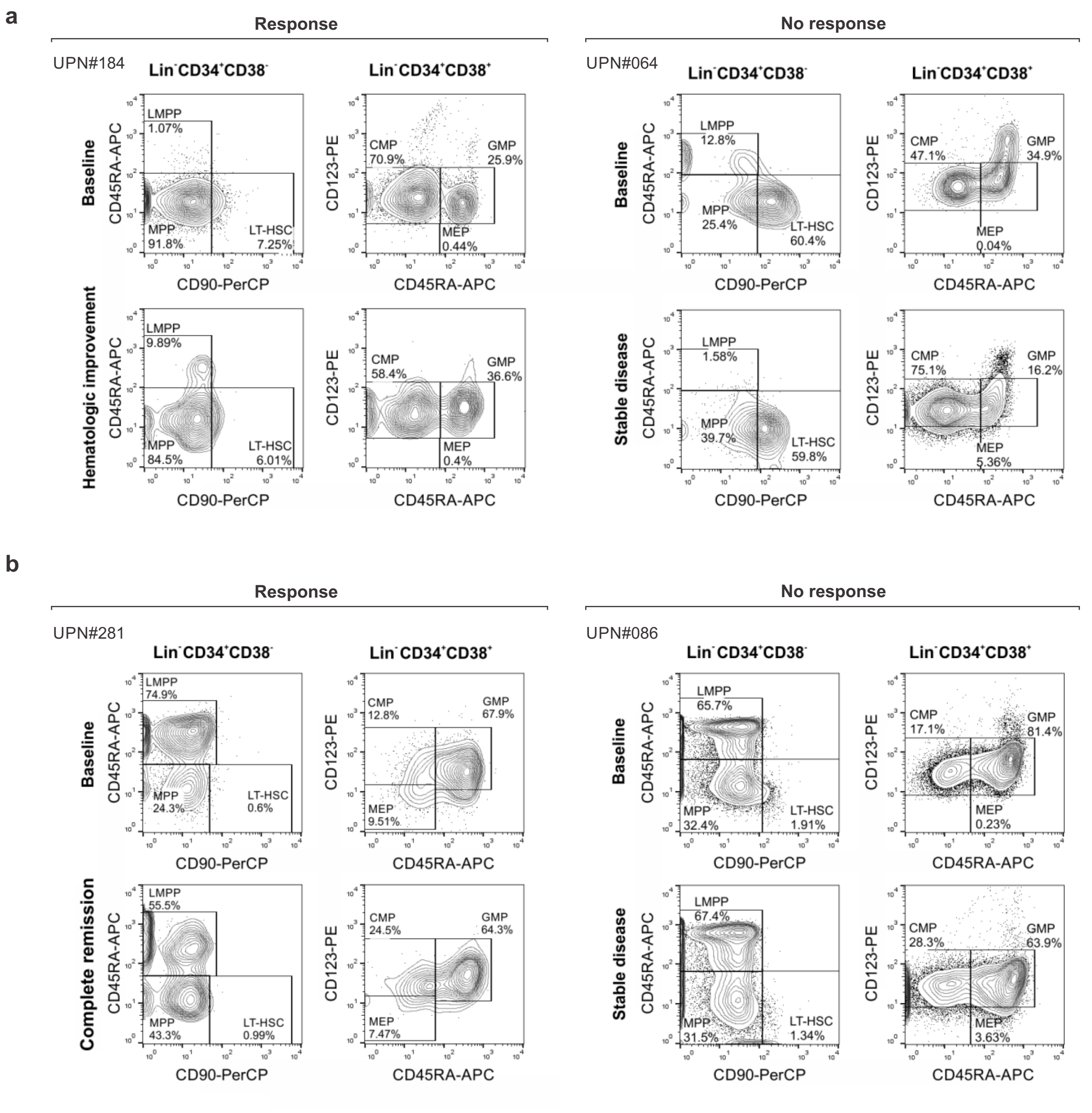

(a) Flow cytometry plots of $Lin^-CD34^+CD38^-$ and $Lin^-CD34^+CD38^+$ HSPC subpopulations in BM samples from two representative "CMP pattern" MDS patients, a responder (left) and a non-responder (right), at baseline (top) and at the time of the best available response or lack of response to HMA therapy, respectively (bottom). **(b)** Flow cytometry plots of $Lin^-CD34^+CD38^-$ and $Lin^-CD34^+CD38^+$ HSPC subpopulations in BM samples from two representative "GMP pattern" MDS patients, a responder (left) and a non-responder (right), at baseline (top) and at the time of the best available response or lack of response to HMA therapy, respectively (bottom).

Supplementary Figure 6. MDS stem cells maintain the disease phenotype during HMA therapy.

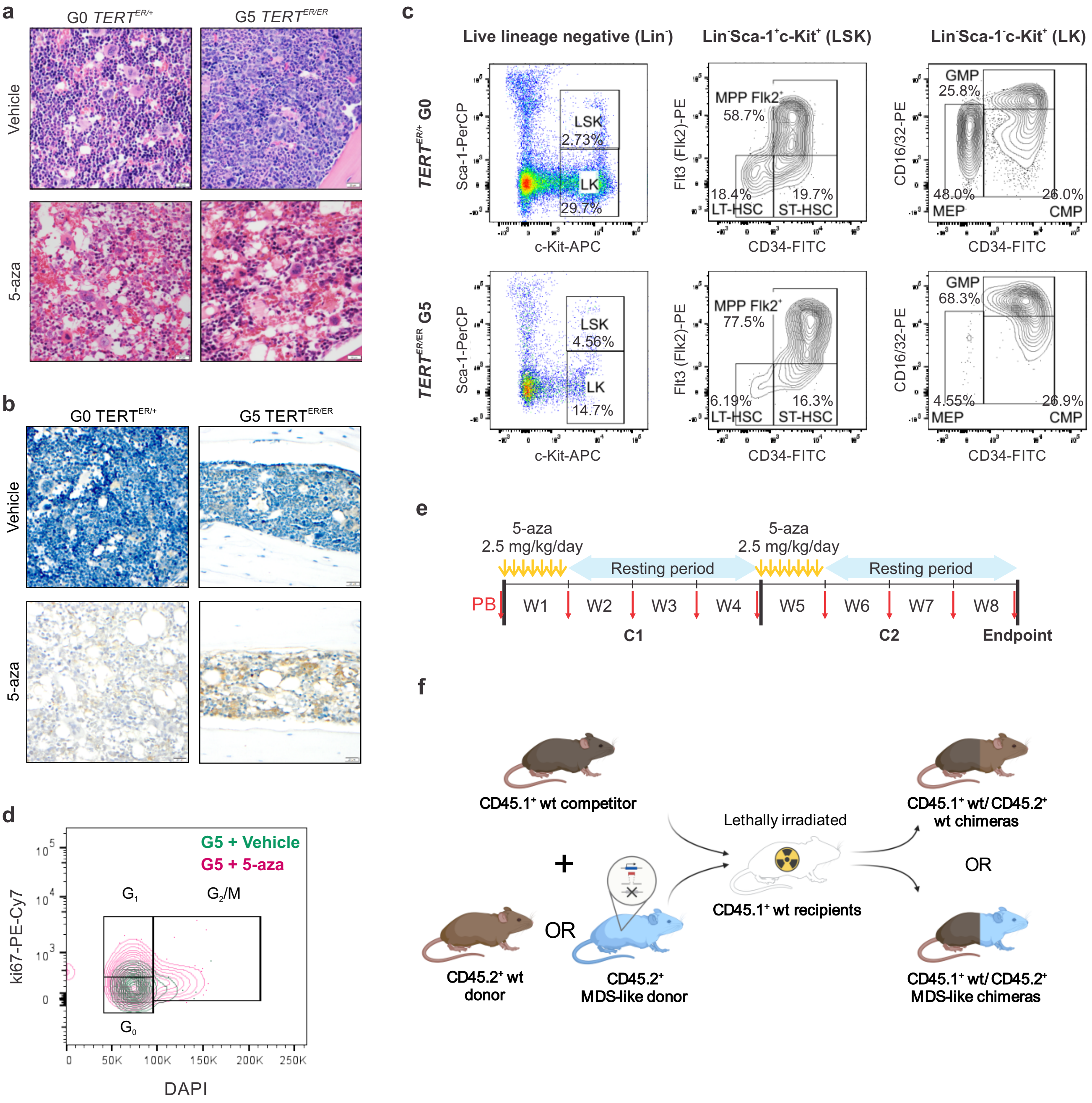

(a) Representative hematoxylin and eosin-stained BM biopsy specimens from G0 *TERT*$^{ER/+}$ and G5 *TERT*$^{ER/ER}$ mice after 1 week of treatment with 5-aza or vehicle. Scale bars, 20 µm (400x magnification). **(b)** Representative anti-cleaved caspase-3-stained BM biopsy specimens from G0 *TERT*$^{ER/+}$ and G5 *TERT*$^{ER/ER}$ mice after 1 week of treatment with 5-aza or vehicle. Scale bars, 20 µm (400x magnification). **(c)** Flow cytometry plots of the LSK and LK populations in BM samples from two representative G0 *TERT*$^{ER/+}$ and G5 *TERT*$^{ER/ER}$ mice. LSK and LK cells were simultaneously analyzed using two different panels in the same BM sample. **(d)** Density plots of cell cycle phases in LT-HSCs from two representative G5 *TERT*$^{ER/ER}$ mice after 1 week of 5-aza treatment. **(e)** Experimental design and 5-aza treatment schedule for the mouse models. Vehicle or 5-aza (2.5 mg/kg/day) were injected intraperitoneally daily during the first week (W) of each treatment cycle (C). BM counts were allowed to recover for the last 3 weeks of each cycle; during this period, PB counts were monitored weekly. BM was analyzed immediately after the end of C2. **(f)** Experimental design of the competitive BM transplantation experiments. A mix of CD45.1^{+} wt cells and CD45.2^{+} donor (MDS-like or control) cells was transplanted into lethally irradiated CD45.1^{+} B6 mice to generate wt/wt and wt/MDS chimeras.

Supplementary Figure 7. Stem cells expand during MDS progression after failure to HMA therapy.

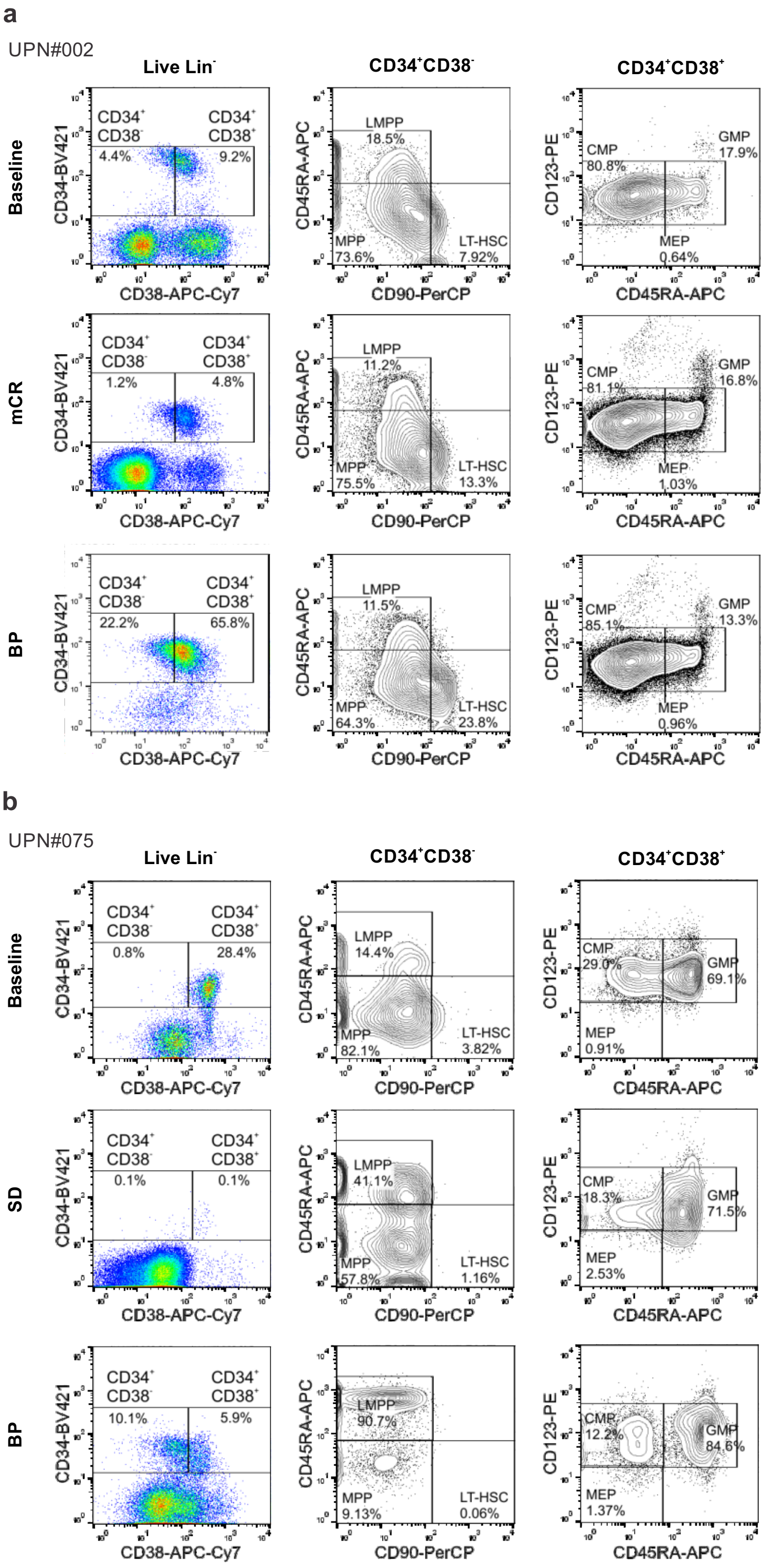

(a) Flow cytometry plots of Lin^-CD34^+ HSPC populations in sequential samples obtained from a representative "CMP pattern" MDS patient at baseline, during HMA therapy, and after therapy failure with BP. **(b)** Flow cytometry plots of Lin^-CD34^+ HSPC populations in sequential samples obtained from a representative "GMP pattern" MDS patient at baseline, during HMA therapy, and after therapy failure with BP. mCR, marrow complete remission; SD, stable disease.

Supplementary Figure 8. Pharmacologically targeting the upregulated survival pathways in MDS stem cells reduces tumor burden.

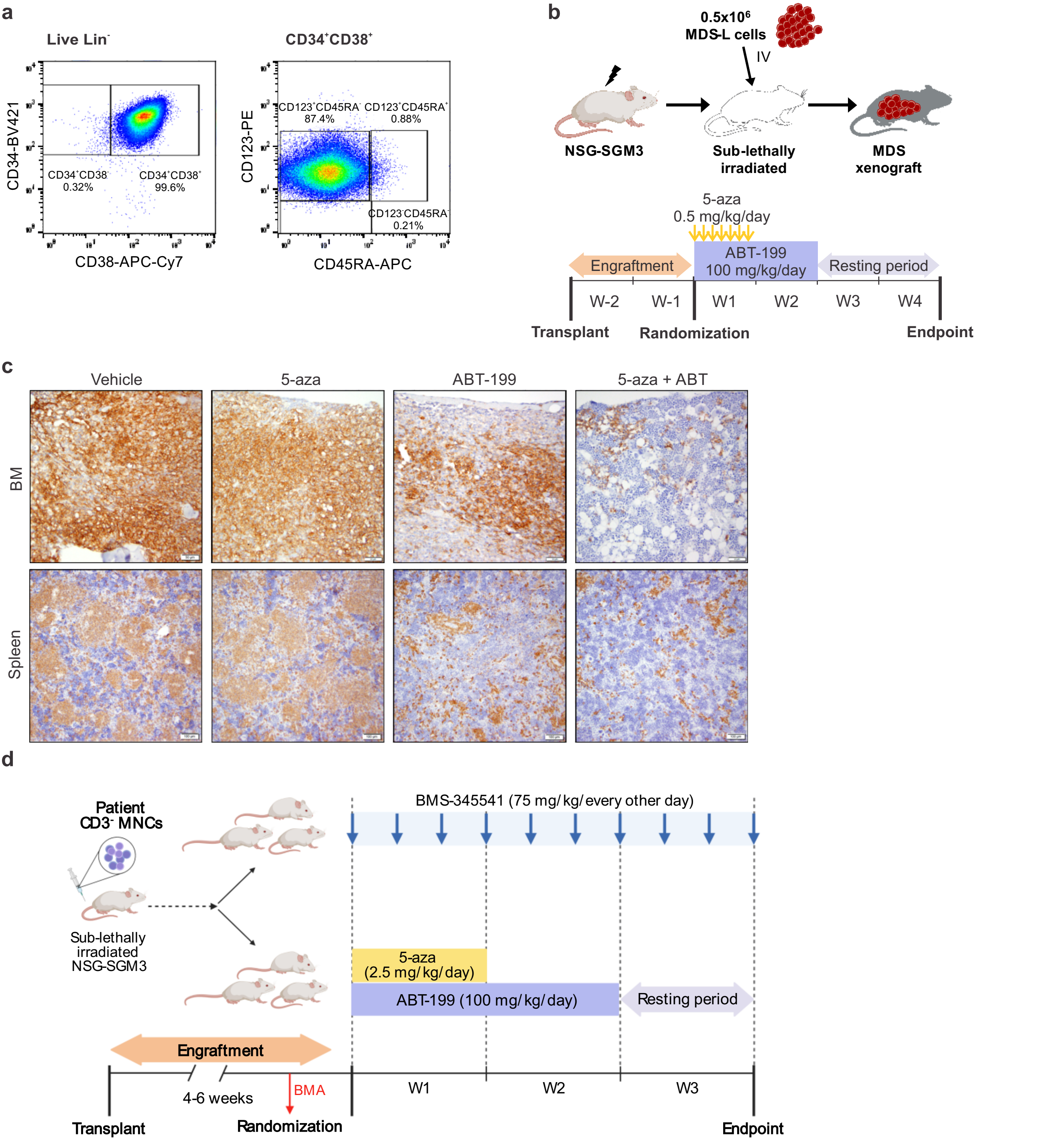

(a) Immunophenotypic profile of MDS-L cells with the HSPC flow cytometry marker panel detailed in Supplementary Table 1. **(b)** Experimental design and treatment schedule for the MDS-L xenograft experiments performed in NSGS mice. **(c)** Representative anti-human CD45 antibody staining of BM (top) and splenic (bottom) biopsy specimens from MDS-L xenografts after one cycle of treatment with ABT-199 and/or 5-aza. Scale bars, 50 µm (200x magnification). **(d)** Experimental design and treatment schedules of xenografts developed by transplanting T cell-depleted BM MNCs from MDS patients with BP into NSGS mice.

Supplementary Figure 9. Pharmacologically targeting the upregulated survival pathways in MDS stem cells halts disease progression.

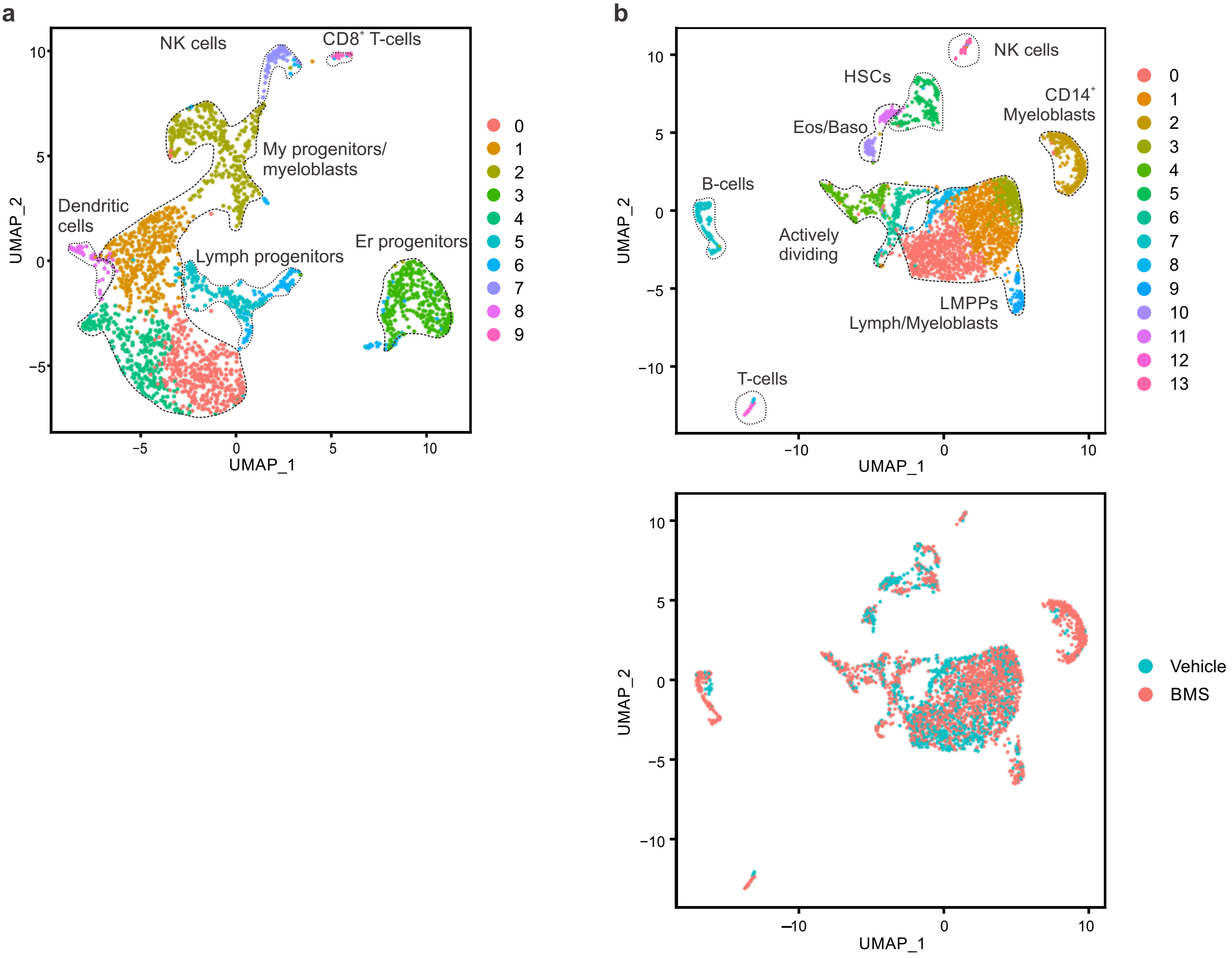

(a) UMAP plot of scRNA-seq data displaying a pool of 2,310 human $CD45^{+}$ cells isolated from two untreated xenografts from the experiment shown in Fig. 3e. Each symbol represents one cell; colors represent different gene expression clusters. Cluster identities are indicated. Er, erythroid; Lymph, lymphoid; My, myeloid. **(b)** UMAP plots of scRNA-seq data displaying a pool of 3,800 human $CD45^{+}$ cells isolated from two of the vehicle-treated (1,208 cells) and two of the BMS-345541-treated (BMS; 2,592 cells) “GMP pattern” xenografts from the experiment shown in Fig. 3e. Each symbol represents one cell. Colors indicate different gene expression clusters (top) or treatment groups (bottom). Cluster identities are indicated on the top plot. Baso, basophil; Eos, eosinophil; Lymph, lymphoid; NK, natural killer.

Supplementary Figure 10. CyTOF surface markers used for cluster annotation.

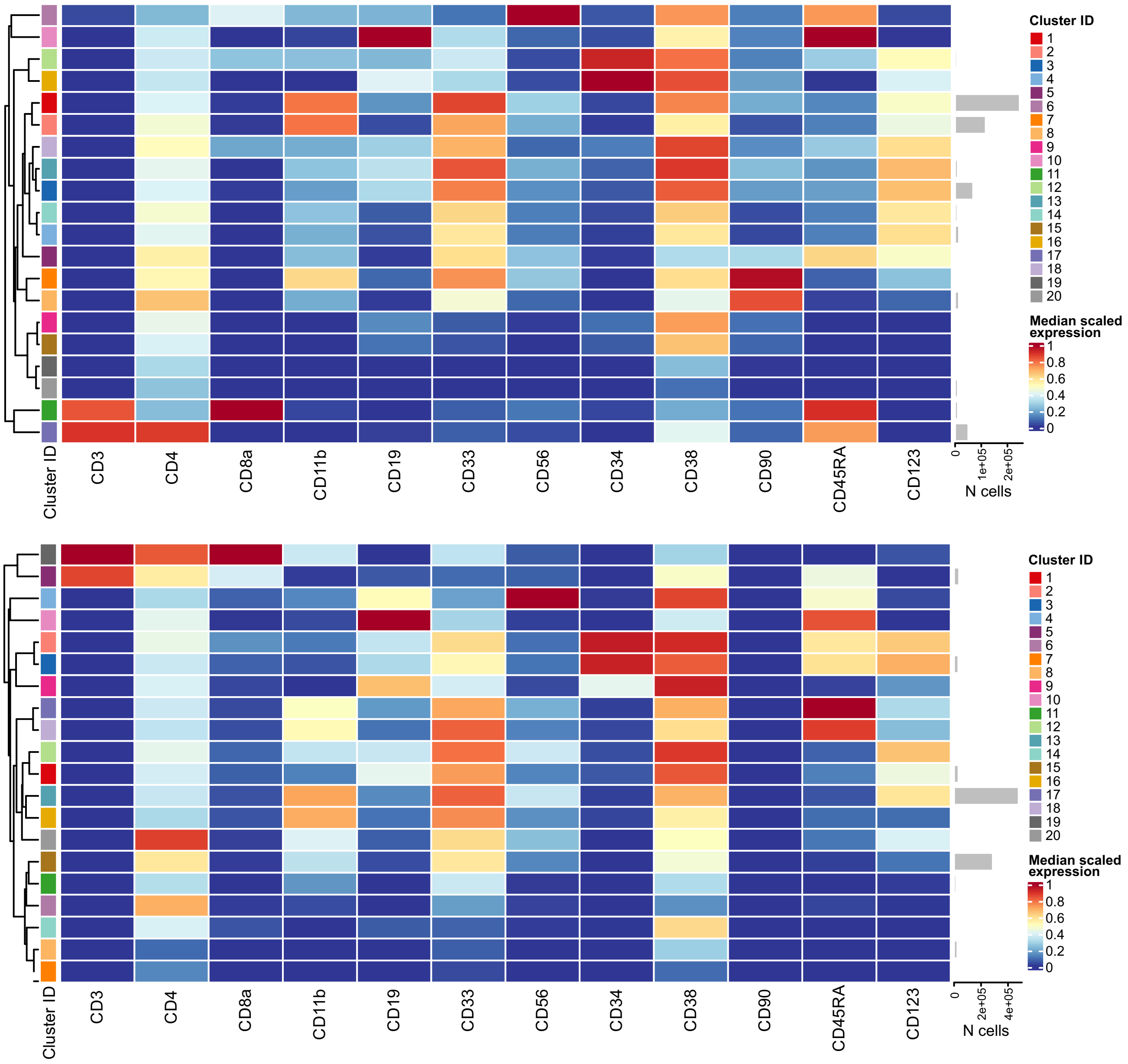

Heatmaps showing the normalized expression of lineage surface markers across the CyTOF clusters of the representative “CMP pattern” (top) or “GMP pattern” (bottom) MDS samples shown in Fig. 4c. The number of cells in each cluster is shown on the right.

Supplementary Figure 11. Venetoclax-based therapy does not target HSCs from "GMP pattern" MDS at BP after HMA therapy failure.

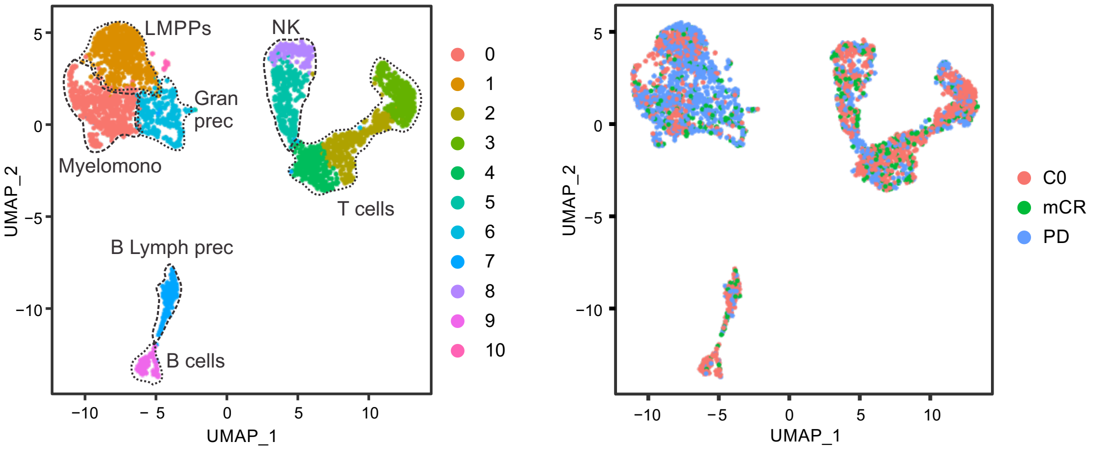

UMAP plots of scRNA-seq data displaying MNCs isolated from sequential samples of the representative "GMP pattern" MDS shown in Fig. 4c. Each symbol represents one cell. Different colors represent the hematopoietic clusters (left) or sample identities (right). Gran, granulocytic; Prec, precursor; Myelomono; myelomonocytic; Lymph, lymphoid; C0, cycle zero; mCR, marrow complete remission; PD, progressive disease.
