# Supplementary material for: Stem cell architecture drives myelodysplastic syndrome progression and predicts response to venetoclax-based therapy
Source: Nat Med. 2022 Mar 3;28(3):557–67. doi: 10.1038/s41591-022-01696-4 (PMC8938266; doi:10.1038/s41591-022-01696-4)
Supplement: Supplementary file 2 — Reporting Summary [file 41591_2022_1696_MOESM2_ESM.pdf]

nature portfolio

Corresponding author(s): Simona Colla

Last updated by author(s): Dec 30, 2021

Reporting Summary

Nature Portfolio wishes to improve the reproducibility of the work that we publish. This form provides structure for consistency and transparency in reporting. For further information on Nature Portfolio policies, see our Editorial Policies and the Editorial Policy Checklist.

Statistics

For all statistical analyses, confirm that the following items are present in the figure legend, table legend, main text, or Methods section.

n/a	Confirmed	
☐	☒	The exact sample size (*n*) for each experimental group/condition, given as a discrete number and unit of measurement
☐	☒	A statement on whether measurements were taken from distinct samples or whether the same sample was measured repeatedly
☐	☒	The statistical test(s) used AND whether they are one- or two-sided<br>*Only common tests should be described solely by name; describe more complex techniques in the Methods section.*
☐	☒	A description of all covariates tested
☐	☒	A description of any assumptions or corrections, such as tests of normality and adjustment for multiple comparisons
☐	☒	A full description of the statistical parameters including central tendency (e.g. means) or other basic estimates (e.g. regression coefficient) AND variation (e.g. standard deviation) or associated estimates of uncertainty (e.g. confidence intervals)
☐	☒	For null hypothesis testing, the test statistic (e.g. *F*, *t*, *r*) with confidence intervals, effect sizes, degrees of freedom and *P* value noted<br>*Give P values as exact values whenever suitable.*
☒	☐	For Bayesian analysis, information on the choice of priors and Markov chain Monte Carlo settings
☐	☒	For hierarchical and complex designs, identification of the appropriate level for tests and full reporting of outcomes
☒	☐	Estimates of effect sizes (e.g. Cohen's *d*, Pearson's *r*), indicating how they were calculated

Our web collection on statistics for biologists contains articles on many of the points above.

Software and code

Policy information about availability of computer code

Data collection	BD FACSDiva, version 8.01 (www. https://www.bdbiosciences.com); IncuCyte S3, version 2017A (www.essenbioscience.com).
Data analysis	FlowJo, version 10.5.3 (www.flowJo.com); GraphPad Prism 8 (www.graphpad.com); R (www.r-project.org); IncuCyte S3 (www.essenbioscience.com); Circos (http://circos.ca); Metascape (https://metascape.org); Seurat v3 (https://satijalab.org/seurat); HTseq software, version 0.11.2 (https://htseq.readthedocs.io/en/master/); FASTQC software, version 0.11.8 (https://www.bioinformatics.babraham.ac.uk/projects/fastqc/); CATALYST, version 1.18.0 (https://bioconductor.org/packages/release/bioc/html/CATALYST.html); FlowSOM, version 2.2 (https://bioconductor.org/packages/release/bioc/html/FlowSOM.html); ConsensusClusterPlus, version 1.58 (https://bioconductor.org/packages/release/bioc/html/ConsensusClusterPlus.html); CytoTREE, version 1.4 (http://www.bioconductor.org/packages/release/bioc/html/CytoTree.html). All of the software used has been included in the “Methods" section of the paper. The R code used for the MDS sample flow cytometry data clustering and classification can be obtained from the authors upon request by email and the resulting mathematical algorithm has been shared in the manuscript.

For manuscripts utilizing custom algorithms or software that are central to the research but not yet described in published literature, software must be made available to editors and reviewers. We strongly encourage code deposition in a community repository (e.g. GitHub). See the Nature Portfolio guidelines for submitting code & software for further information.

Data

Policy information about availability of data

All manuscripts must include a data availability statement. This statement should provide the following information, where applicable:

- Accession codes, unique identifiers, or web links for publicly available datasets
- A description of any restrictions on data availability
- For clinical datasets or third party data, please ensure that the statement adheres to our policy

Data sets generated in this study by RNA-seq in HSCs and progenitors and scRNA-seq are publicly accessible at GEO under GSE178840 and GSE137429, respectively. All can be accessed from the super-series GSE136816. Data generated by DNA-seq are accessible at EGA under S00001003867, S00001003869, and S00001003868 and at BioProject under PRJNA737460.
The human genome (hg19) datatbase is accessible at https://www.ncbi.nlm.nih.gov/assembly/GCF_000001405.13/.

Field-specific reporting

Please select the one below that is the best fit for your research. If you are not sure, read the appropriate sections before making your selection.

☒ Life sciences ☐ Behavioural & social sciences ☐ Ecological, evolutionary & environmental sciences

For a reference copy of the document with all sections, see nature.com/documents/nr-reporting-summary-flat.pdf

Life sciences study design

All studies must disclose on these points even when the disclosure is negative.

Sample size

In all our human studies, no prior sample-size calculations were possible to predetermine sample size; human samples were used based on availability of fresh or banked samples and selected based only on the stage of MDS disease (diagnosis, progressive disease), prior therapies (no therapies other than supportive care) and current therapy (hypomethylating agents and/or venetoclax). The same applies to IHC analyses, where no prior sample-size calculations were possible and samples were obtained based on availability of paraffin blocks from patients of interest. A more thorough description of our patient selection criteria is in the “Methods” section of the manuscript and in the “Human research participants” section below, and a summary of their clinical characteristics is in Supplementary Table 2. In scRNA-seq and CyTOF experiments, the number of subjects is particularly low owing to the difficulty and elevated cost of these techniques, which is why we only used them to validate other results. Sample size was considered to be sufficient in both sets of experiments in sight of the consistency of the data with our previous hypothesis-generating results.
In mouse studies, we estimated a number of 7 samples per group to be sufficient to identify differences between groups with 80% power, assuming a large effect size. Thus, we generally began experiments with n>=7 per experimental group and in most cases we performed the experiment in duplicate or even in triplicate. In a few cases (as in the setting of transplantation), mice died during the experiment and the number was reduced to 4-6 mice per group. In those cases, we considered the results as valid only if they were consistent with other experiments and/or if differences between experimental groups were clear and statistically significant.
In cell line experiments other than mouse xenotransplants (see above), specifically in a Western blot and flow cytometric evaluation performed in MDS-L cells, sample size was not pre-calculated as both experiments were performed in untreated cells to confirm intrinsic properties of the cell line (no experimental conditions or variables were applied). Each of these experiments were performed twice with different batches of the cell line to confirm consistency across passages.

Data exclusions

In mouse studies, a few mice were excluded from endpoint analyses because they developed pathologies unrelated to the experimental design, such as abnormal blood or bone marrow counts that were indicative of an irradiation-induced disease (e.g. host-derived lymphocytic malignancy) or signs of infection (in immunodeficient mice). In cases where drugs were administered by oral gavage, we excluded mice in which we found signs of esophagus perforation during necropsy, on the basis of the assumptions that the drug’s absorbance may have been reduced and inflammation secondary to the perforation may have altered the bone marrow of the mice. Any mouse exclusion was blinded.

Replication

For replicated experiments, the number of replicates is indicated in the figure legends. Some mouse competitive transplant experiments were performed only once; however, we validated our findings in 5 different genetic mouse models.

Randomization

All mouse experiments were randomized. For those performed in transgenic mice, we randomly assigned equal number of males and female into each group. For transplantation experiments, we assessed the level of engraftment (% chimerism) of mouse or human cells and randomized the mice into experimental groups of similar mean % chimerism.
Randomization does not apply to experiments performed in human samples, as these needed to be pre-selected on the basis of the patient diagnosis, past and current therapies as explained above.

Blinding

Experiments performed in transgenic mice were randomized blindly. Blinding does not apply to randomization in mouse transplantation experiments (including xenograft mice) because, as explained above, the percent engraftment of mouse or human cells had to be known beforehand to exclude failed grafts.
Blinding was not possible in human sample selection for the same reasons explained above for the lack of randomization.
Experimental data analysis was performed blindly in all experiments. Blinding was not possible in clinical data analysis as all patients characteristics have to be known for this type of analysis.

Reporting for specific materials, systems and methods

We require information from authors about some types of materials, experimental systems and methods used in many studies. Here, indicate whether each material, system or method listed is relevant to your study. If you are not sure if a list item applies to your research, read the appropriate section before selecting a response.

Materials & experimental systems

n/a	Involved in the study
☐	☒ Antibodies
☐	☒ Eukaryotic cell lines
☒	☐ Palaeontology and archaeology
☐	☒ Animals and other organisms
☐	☒ Human research participants
☒	☐ Clinical data
☒	☐ Dual use research of concern

Methods

n/a	Involved in the study
☒	☐ ChIP-seq
☐	☒ Flow cytometry
☒	☐ MRI-based neuroimaging

Antibodies

Antibodies used

A detailed antibody list that includes vendor, clone information and dilution is included in the "Methods" section of the paper. The antigen panels used in each flow cytometry analysis are detailed in Supplementary Tables 1 and 10. Flow cytometry analyzer and sorter settings for those antibodies are indicated in Supplementary Table 9. The antibodies used in the CyTOF panel are indicated in Supplementary Table 11. Suppliers, catalog number, clone and dilution information for all antibodies is provided below:
Use, Species, Antigen, Conjugate, Clone, Dilution, Supplier, Catalog #
Flow cytometry, Human, CD2, FITC, RPA-2.10, 1:20, BD Biosciences, 555326
Flow cytometry, Human, CD3, FITC, SK7, 1:10, BD Biosciences, 349201
Flow cytometry, Human, CD4, FITC, S3.5, 1:20, Thermo Fisher, MHCD0401
Flow cytometry, Human, CD7, FITC, 6B7, 1:20, BioLegend, 343104
Flow cytometry, Human, CD10, FITC, SJ5-1B4, 1:20, Leinco Technologies, C139
Flow cytometry, Human, CD11b, FITC, ICRF44, 1:20, Thermo Fisher, 11-0118-42
Flow cytometry, Human, CD14, FITC, MϕP9, 1:20, BD Biosciences, 347493
Flow cytometry, Human, CD19, FITC, SJ25C1, 1:10, BD Biosciences, 340409
Flow cytometry, Human, CD20, FITC, 2H7, 1:10, BD Biosciences, 555622
Flow cytometry, Human, CD33, FITC, P67.6, 1:20, Thermo Fisher, 11-0337-42
Flow cytometry, Human, CD56, FITC, B159, 1:40, BD Biosciences, 562794
Flow cytometry, Human, CD66b, FITC, G10F5, 1:20, BD Biosciences, 561927
Flow cytometry, Human, CD235a, FITC, HIR2, 1:40, BD Biosciences, 559943
Flow cytometry, Human, CD45RA, APC, HI100, 1:10, Tonbo , 20-0458-T100
Flow cytometry, Human, CD34, BV421, 581, 1:20, BD Biosciences, 562577
Flow cytometry, Human, CD123, PE, 9F5, 1:20, BD Biosciences, 555644
Flow cytometry, Human, CD90, Per-CP, 5E10, 1:10, Thermo Fisher, 45-0909-42
Flow cytometry, Human, CD38, APC, HIT2, 1:20, BioLegend, 303534
Flow cytometry, N/A, Streptavidin, APC-Cy7, N/A, 1:100, BD Biosciences, 554063
Flow cytometry, Mouse, CD34, FITC, RAM34, 1:20, Thermo Fisher, 11-0341-85
Flow cytometry, Mouse, Sca-1, Per-CP, D7, 1:100, Thermo Fisher, 45-5981-82
Flow cytometry, Mouse, CD135/Flt3, PE, A2F10, 1:40, Thermo Fisher, 12-1351-82
Flow cytometry, Mouse, CD16/CD32, PE, 93, 1:200, Thermo Fisher, 12-0161-82
Flow cytometry, Mouse, CD117/c-Kit, APC, 2B8, 1:200, BD Biosciences, 553356
Flow cytometry, Mouse, CD117/c-Kit, PE-Cy7, 2B8, 1:200, Thermo Fisher, 25-1171-81
Flow cytometry, Mouse, CD117/c-Kit, BV421, 2B8, 1:200, BD Biosciences, 562609
Flow cytometry, Mouse, CD45.2, BV605, 104, 1:20, BioLegend, 109841
Flow cytometry, Mouse, Ki67, APC, SolA15, 1:20, Thermo Fisher, 17-5698-82
Flow cytometry, Mouse, CD45R/B220, APC-Cy7, RA3 6B2, 1:100, BD Biosciences, 552094
Flow cytometry, Mouse, CD45.1, FITC, A20, 1:100, Thermo Fisher , 11-0453-82
Flow cytometry, Mouse, CD45.2, PE, 104, 1:40, Thermo Fisher , 12-0454-82
Flow cytometry, Mouse, Gr-1/Ly-6G/6C, Per-CP, RB6-8C5, 1:200, Thermo Fisher , 45-5931-80
Flow cytometry, Mouse, CD3e, APC, 145-2C11, 1:100, Thermo Fisher , 17-0031-82
Flow cytometry, Mouse, CD45, FITC, 30-F11, 1:20, BioLegend, 103108
Flow cytometry, Human , CD45, PE, HI30, 1:10, BD Biosciences, 555483
IHC, Mouse, Cleaved caspase-3, N/A, N/A, 1:100, Biocare Medical, 229
IHC, Human, CD45, N/A, D9M8I, 1:200, Cell Signaling, 13917
IHC, Human, BCL2, N/A, 124, 1:500, Dako, M088729-2
IHC, Human , p-p65, N/A, S536, 1:750, Abcam, ab86299
Western blot, Human, BCL2, N/A, 124, 1:1000, Dako, M088729-2
Western blot, Human , p65, N/A, D14E12, 1:1000, Cell Signaling, 8242S
Western blot, Human , Phospho-p65, N/A, 93H1, Ser536, 1:1000, Cell Signaling, 3033S
Western blot, Human, Vinculin, N/A, hVIN-1, 1:2000, Sigma-Aldrich, V9131-100UL
Western blot, Mouse, Mouse IgG, HRP, N/A, 1:2000, Kindle Biosciences, R1005
Western blot, Rabbit, Rabbit IgG, HRP, N/A, 1:2000, Kindle Biosciences, R1006
CyTOF, Human, CD45, Y 89, HI30, 2.5ug/ml, Biolegend, 304002

CyTOF, Human, N/A, Pd 102, N/A, N/A, Fluidigm , 201060
CyTOF, Human, N/A, Pd 104, N/A, N/A, Fluidigm, 201060
CyTOF, Human, N/A, Pd 105, N/A, N/A, Fluidigm, 201060
CyTOF, Human, N/A, Pd 106, N/A, N/A, Fluidigm, 201060
CyTOF, Human, N/A, Pd 108, N/A, N/A, Fluidigm, 201060
CyTOF, Human, N/A, Pd 110, N/A, N/A, Fluidigm, 201060
CyTOF, Human, CD11b, Cd 111, ICRF44, 2.5ug/ml, Biolegend, 301337
CyTOF, Human, CLA, Cd 112, HECA-452, 1.25ug/ml, BD Biosciences, 555947
CyTOF, Human, CD8, In 113, RPA-T8, 2ug/ml, Biolegend, 301053
CyTOF, Human, CD47, Cd 114, CC2C6, 2.5ug/ml, Biolegend, 323102
CyTOF, Human, CD3, In 115, UCHT1, 2ug/ml, Biolegend, 300443
CyTOF, Human, p-H2AX, Cd 116, 2F3, 2.5ug/ml, Biolegend, 613402
CyTOF, Human, N/A, I* 127, N/A, 10uM, Thermo Fisher, 122350010
CyTOF, Human, CD36, La 139, 5-271, 0.5ug/ml, Biolegend, 336215
CyTOF, Human, Ubiquitin, Ce 140, EPR8830, 2ug/ml, Abcam, ab134953
CyTOF, Human, BCL-XL, Pr 141, 54H6, 2.5ug/ml, Cell Signaling, Custom
CyTOF, Human, CD68, Nd 142, KP1, 1ug/ml, Thermo Fisher, 14-0688-82
CyTOF, Human, CD56, Nd 143, NCAM16.2, 1.5ug/ml, BD Biosciences, 559043
CyTOF, Human, BCL2, Nd 144, 100, 2ug/ml, Biolegend, 658702
CyTOF, Human, CD123, Nd 145, 6H6, 2.5ug/ml, Biolegend, 306027
CyTOF, Human, BIM, Nd 146, C34C5, 2.5ug/ml, Cell Signaling, Custom
CyTOF, Human, p-4EBP1, Sm 147, 236B4, 2.5ug/ml, Cell Signaling, Custom
CyTOF, Human, CD34, Nd 148, 581, 2ug/ml, Biolegend, 343531
CyTOF, Human, BAD, Sm 149, 48/Bad, 2.5ug/ml, BD Biosciences, Custom
CyTOF, Human, p-STAT5(Y694), Nd 150, 47, 2.5ug/ml, BD Biosciences, Custom
CyTOF, Human, CLL-1, Eu 151, 50C1, 1.25ug/ml, Biolegend, 353602
CyTOF, Human, p-STAT3(Y705), Sm 152, 4/P-STAT3, 2.5ug/ml, BD Biosciences, Custom
CyTOF, Human, CD45RA, Eu 153, HI100, 2ug/ml, Biolegend, 304102
CyTOF, Human, Ki-67, Sm 154, 20Raj1, 1.25ug/ml, Thermo Fisher, 14-5699-82
CyTOF, Human, PD-L1, Gd 155, 29E.2A3, 2.5ug/ml, Biolegend, 329719
CyTOF, Human, CD38, Gd 156, HIT2, 1.25ug/ml, Biolegend, 303535
CyTOF, Human, CD19, Gd 157, HIB19, 2ug/ml, Biolegend, 302247
CyTOF, Human, CD33, Gd 158, WM53, 2ug/ml, Biolegend, 303419
CyTOF, Human, p-AKT(S473), Tb 159, M89-61, 2.5ug/ml, BD Biosciences, Custom
CyTOF, Human, YTHDF2, Gd 160, Polyclonal, 2ug/ml, Proteintech (Rosemont, IL), 24744-1-AP
CyTOF, Human, p-GSK-3(S21/S9), Dy 161, Polyclonal, 2.5ug/ml, R&D Systems, AF1590
CyTOF, Human, Survivin, Dy 162, Polyclonal, 2.5ug/ml, R&D Systems, AF6471
CyTOF, Human, c-MYC, Dy 163, D84C12, 2.5ug/ml, Cell Signaling, Custom
CyTOF, Human, MDM2, Dy 164, D-12, 2.5ug/ml, Santa Cruz, Custom
CyTOF, Human, TP53, Ho 165, DO-7, 2ug/ml, BD Biosciences, 554294
CyTOF, Human, C-KIT, Er 166, 104D2, 2ug/ml, Biolegend, 313202
CyTOF, Human, p-ERK1/2 (p44/42), Er 167, D13.14.4.E, 2.5ug/ml, Cell Signaling, Custom
CyTOF, Human, NOXA, Er 168, 114C307.1, 2.5ug/ml, Abcam, 633602
CyTOF, Human, p-MEK1/2(Ser217/219), Tm 169, 41G9, 2.5ug/ml, Cell Signaling, Custom
CyTOF, Human, PUMA, Er 170, EP512Y, 2.5ug/ml, Abcam, AB186917
CyTOF, Human, CD90, Yb 171, 5E10, 2.5ug/ml, Biolegend, 328102
CyTOF, Human, p-S6(S240/244), Yb 172, D68F8, 2ug/ml, Cell Signaling, Custom
CyTOF, Human, BAX, Yb 173, 2D2, 1.5ug/ml, Biolegend, 633602
CyTOF, Human, p-FLT3(Tyr589/591), Yb 174, 30D4, 2.5ug/ml, Cell Signaling, Custom
CyTOF, Human, CXCR4, Lu 175, 12G5, 1.5ug/ml, Biolegend, 306502
CyTOF, Human, MCL1, Yb 176, D2W9E, 2ug/ml, Cell Signaling, Custom
CyTOF, Human, DNA, Ir 191, N/A, 100nm, Fluidigm, 201192B
CyTOF, Human, DNA, Ir 193, N/A, 100nm, Fluidigm, 201192B
CyTOF, Human, CD4, Pt 194, RPA-T4, 2ug/ml, Biolegend, 300502
CyTOF, Human, HLA-ABC, Pt 195, W6/32, 1.5ug/ml, Biolegend, 311402
CyTOF, Human, N/A, Pt† 196, N/A, 500nm, Enzo (New York, NY), ALX-400-040-M050
CyTOF, Human, HLA-DR, Pt 198, L243, 2ug/ml, Biolegend, 307602
CyTOF, Human, H3K27, Bi 209, C36B11, 1ug/ml, Cell Signaling, Custom

Validation

Every antibody used in this study had been previously validated by the manufacturer. In addition:
All antibodies used in human and mouse flow cytometry experiments had been previously validated by other groups (Will et al. Blood 2012; Pang et al. Proc Natl Acad Sci USA 2013) and by us (Colla et al. Cancer Cell 2015; Thongon et al. Nat Commun. 2021), and all our flow cytometry experiments were performed using single-color and "fluorescence-minus-one" controls as indicated in the "Methods" section of the paper.
Antibodies used in Western blots and immunohistochemistry were validated by the manufacturers (anti-huCD45, https://www.cellsignal.com/products/primary-antibodies/cd45-intracellular-domain-d9m8i-xp-rabbit-mab/13917 and Sun et al. Stem Cells Int 2019, Morales et al. Sci Rep. 2019; anti-huBCL2, https://www.agilent.com/en/product/immunohistochemistry/antibodies-controls/primary-antibodies/bcl2-oncoprotein-(concentrate)-76553 and Pezzella et al. Am J Pathol. 1990; anti-huPhosho-p65 [IHC], https://www.abcam.com/nf-kb-p65-phospho-s536-antibody-ab86299.html and He et al. Oncogene 2021; anti-hup65, https://www.cellsignal.com/products/primary-antibodies/nf-kb-p65-d14e12-xp-rabbit-mab/8242 and Omi et al. Oncol Rep 2021; anti-huPhospho-p65 [WB], https://www.cellsignal.com/products/primary-antibodies/phospho-nf-kb-p65-ser536-93h1-rabbit-mab/3033 and Oikawa et al. Front Immunol. 2020; anti-huVinculin, https://www.sigmaaldrich.com/US/en/product/sigma/v9131 and Song et al. Nat Commun. 2021) and we further validated them using positive and negative controls from the corresponding species. In Western blot experiments, the cell lines HL60 and JJN3 were used as positive controls and are shown in every blot.
Antibodies used in CyTOF had been previously validated by others (Han et al. Cytometry A. 2015; Zeng et al. Methods Mol Biol. 2017;

Muftuoglu et al. Sci Rep. 2021).

Eukaryotic cell lines

Policy information about cell lines

Cell line source(s)	The MDS-L cell line was donated by Dr. Kaoru Tohyama (Department of Laboratory Medicine, Kawasaki Medical School, Okayama, Japan).
Authentication	The identity of the MDS-L line was confirmed by short tandem repeat DNA fingerprinting at MD Anderson's Characterized Cell Line Core Facility.
Mycoplasma contamination	Cells were tested positive for mycoplasma contamination at MD Anderson's Characterized Cell Line Core Facility.
Commonly misidentified lines (See ICLAC register)	No commonly misidentified lines were used in the study.

Animals and other organisms

Policy information about studies involving animals; ARRIVE guidelines recommended for reporting animal research

Laboratory animals	Mice were maintained under specific-pathogen-free conditions at MD Anderson and housed in a barrier facility at 25°C under ambient oxygen conditions in a 12-h light/12-h dark cycle under 50% humidity. All animal experiments were performed with the approval of MD Anderson's Institutional Animal Care and Use Committee. All animal studies used 12 to 16-week-old mice unless otherwise indicated.<br>Mice with short telomeres develop MDS-like phenotype independently of the gender. Thus, both genders were equally distributed in our experimental cohorts. Recipient sex plays a critical role in the engraftment and proliferation of human HSCs. Specifically, the use of female NSGS mice is far superior to that of their male counterparts in experiments that involve the engraftment and detection of single human HSCs (Notta F et al. Blood 2010). Thus, only female recipient NSGS mice were used to develop patient-derived xenografts.<br>We generated heterozygous G0 TERTER/+ and late-generation homozygous G5 TERTER/ER mice in-house using a standard breeding protocol. We generated Vav-Cre/Tet2L/L mice by crossing Vav-Cre mice with Tet2L/L mice, and generated Mx1-Cre/Srsf2P95H+/- mice by crossing Mx1-Cre mice with Srsf2P95H+/- mice (all from The Jackson Laboratory, Bar Harbor, ME). We further crossed Mx1-Cre/Srsf2P95H+/- mice with Runx1L/+ mice (The Jackson Laboratory) to obtain Srsf2P95H+/-/Runx1L/+ mice. U2AF1S34F/rtRA mice and C57BL/6J (B6) mice and NSGS (NSG-SGM3) mice were obtained directly from The Jackson Laboratory.
Wild animals	No wild animals were used in the study.
Field-collected samples	No field collected samples were used in the study.
Ethics oversight	All animal experiments were performed with the approval of MD Anderson's Institutional Animal Care and Use Committee.

Note that full information on the approval of the study protocol must also be provided in the manuscript.

Human research participants

Policy information about studies involving human research participants

Population characteristics	Samples were collected from patients with MDS and secondary AML who had not received any therapy other than supportive care (only MDS) or who were receiving (only MDS) or had received and failed (MDS and AML) therapy with hypomethylating agents as single agents, with the exception of combinations with the cytidine deaminase inhibitor E7727 (cedazuridine). In cases who had received supportive care, samples were collected more than 1 week after the completion of growth factor therapy. The clinical characteristics of the MDS patients at diagnosis are shown in Supplementary Table 2. Responses to HMA therapy and clinical outcomes are shown in Supplementary Table 8.<br>Myelodysplastic syndromes occur at the same rates in males and females and is more frequent in individuals over the age of 50. In agreement with this long-standing observation, we did not detect significant differences in sex or age distribution in the two groups of MDS patients. The median age was 70 years old.
Recruitment	Fresh bone marrow samples were routinely collected for research purposes from patients referred to the Department of Leukemia at MD Anderson Cancer Center who had previously signed an informed consent. Frozen bone marrow samples were obtained from the Leukemia Specimen Bank, which houses routinely-collected research samples, and from the University of Parma. In all cases, we selected the samples for our study based on the selection criteria indicated above.
Ethics oversight	All samples were obtained after the approval by the corresponding Institutional Review Boards at MD Anderson Cancer Center (Houston, TX, US) and University of Parma (Parma, Italy) and in accordance with the Declaration of Helsinki.

Note that full information on the approval of the study protocol must also be provided in the manuscript.

Flow Cytometry

Plots

Confirm that:

☒ The axis labels state the marker and fluorochrome used (e.g. CD4-FITC).

☒ The axis scales are clearly visible. Include numbers along axes only for bottom left plot of group (a 'group' is an analysis of identical markers).

☒ All plots are contour plots with outliers or pseudocolor plots.

☒ A numerical value for number of cells or percentage (with statistics) is provided.

Methodology

Sample preparation	Sample preparation details are extensively described in the "Methods" section of the paper. Briefly, bone marrow (BM) aspirates were collected from research subjects following standard clinical procedures. BM mononuclear cells were isolated from each sample using the standard gradient separation approach with Ficoll-Paque PLUS (GE Healthcare Lifesciences, Pittsburgh, PA). For cell sorting, MNCs were pre-enriched using magnetic sorting with microbead kits for the specific antigens (Miltenyi Biotec, San Diego, CA) and further purified by fluorescence-activated cell sorting (FACS). Mononuclear cells or pre-enriched populations were washed with PBS/10% FBS and stained with the corresponding antibody cocktails.
Instrument	BD LSR Fortessa or BD Influx Cell Sorter (BD Biosciences).
Software	BD FACSDiva, version 8.01 (www. https://www.bdbiosciences.com)
Cell population abundance	Every cell sorting experiment was performed using a double step purification protocol (magnetic bead enrichment followed by FACS). In preliminary validation experiments in which we re-run sorted samples to evaluate if the purification was successful, the purity of double-sorted HSPC populations was over 95%. Given that in the experiments included in the paper (FACS purification for WES, RNA-seq or scRNA-seq) we sorted very low numbers from rare HSPC populations (25-15,000 cells, depending on the experiment), it was impossible to validate each individual experiment.
Gating strategy	We have extensively explained our immunophenotic strategy to characterize both stem and progenitor cells in Supplementary Tables 1 and 10. We showed examples of our strategy in Supplementary figures 5-8. As we stated in our manuscript, our analyses used previously validated stem and progenitor markers.

☒ Tick this box to confirm that a figure exemplifying the gating strategy is provided in the Supplementary Information.
