# Supplementary material for: Stem cell architecture drives myelodysplastic syndrome progression and predicts response to venetoclax-based therapy
Source: Nat Med. 2022 Mar 3;28(3):557–67. doi: 10.1038/s41591-022-01696-4 (PMC8938266; doi:10.1038/s41591-022-01696-4)

Extended Data Fig. 9d. Western blot of BCL2 in MDS-L cells

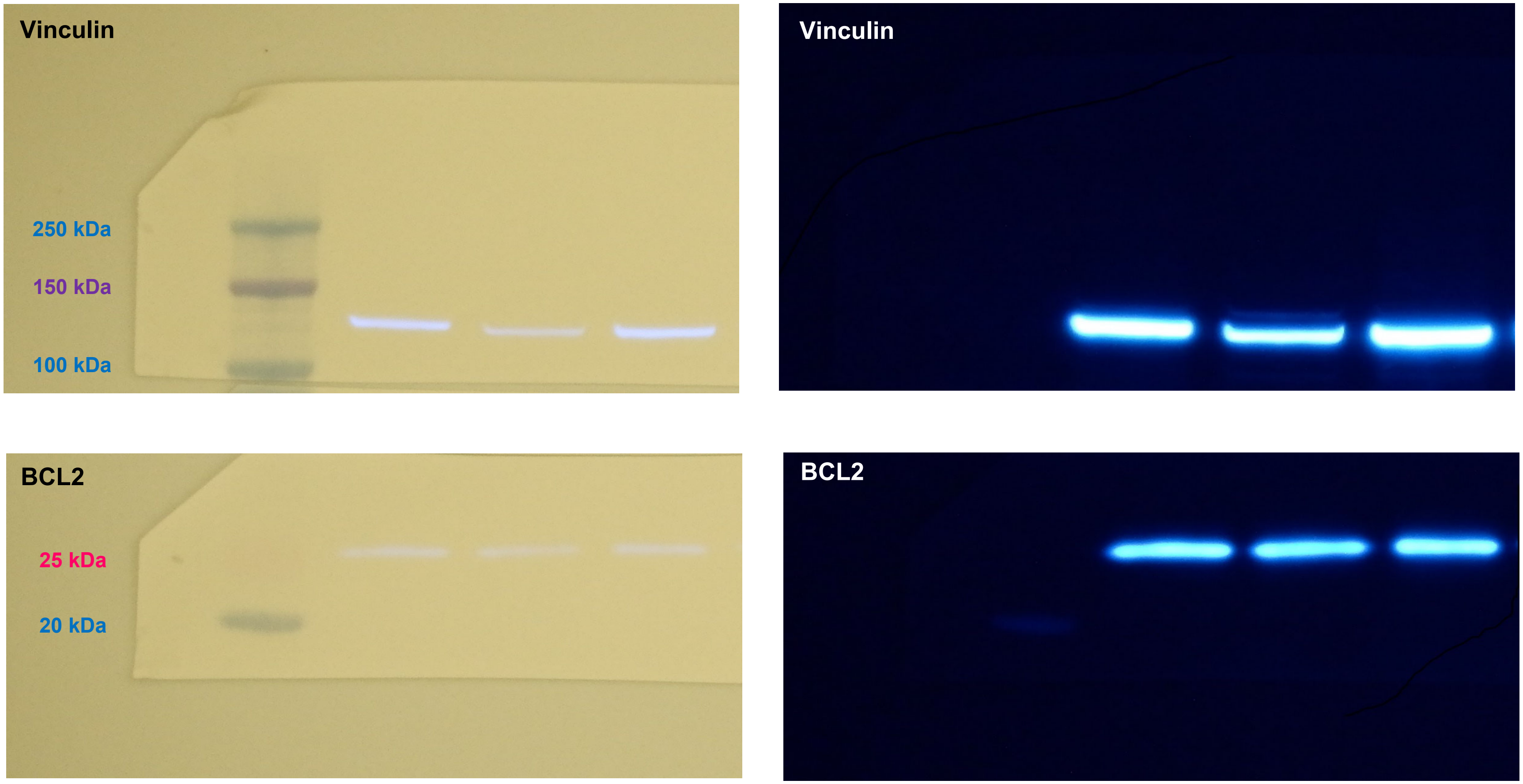

Extended Data Fig. 9j. Western blot of p65, p-p65 and BCL2 in a "GMP pattern" AML xenograft

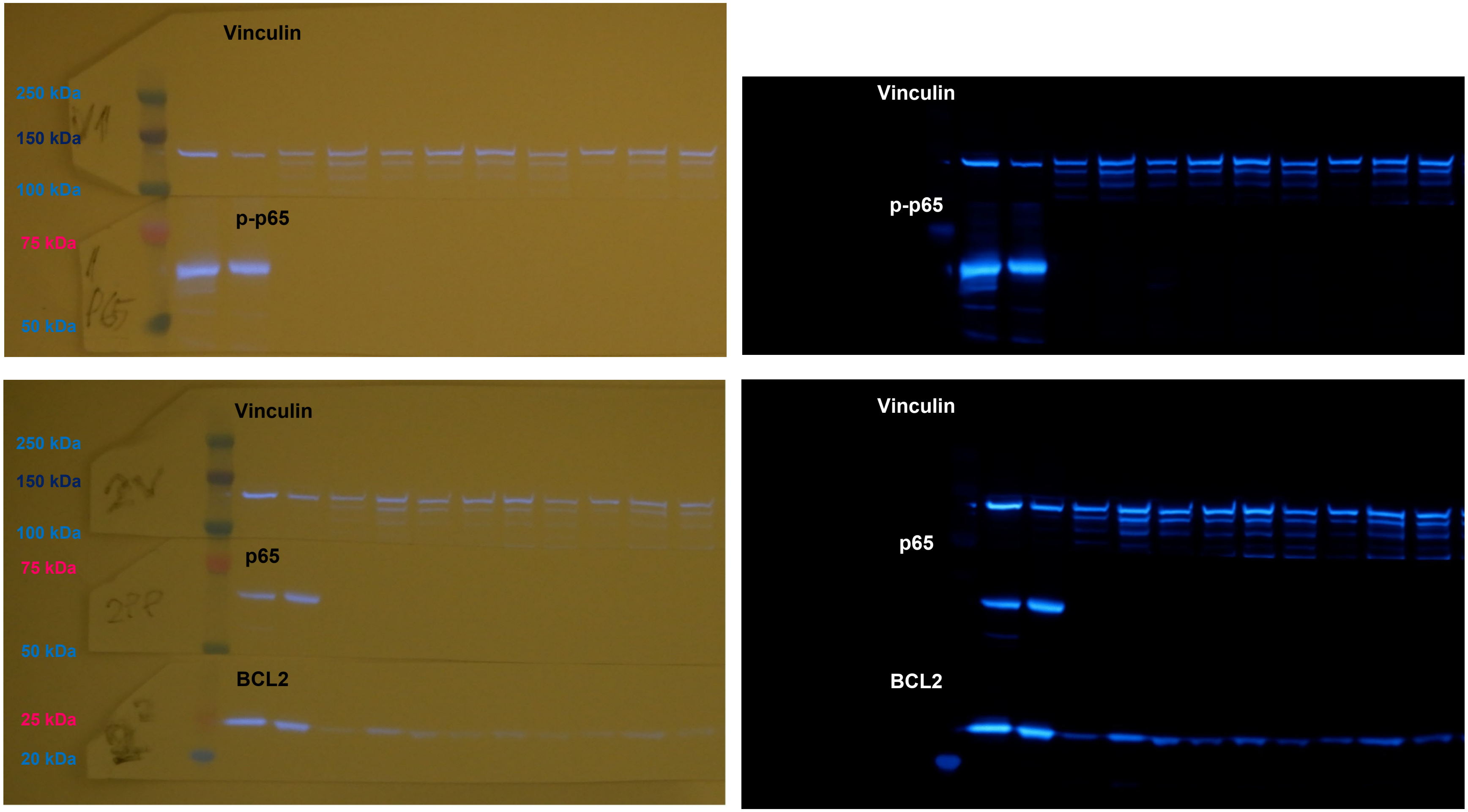

Supplement: Source Data Extended Data Fig. 9 — Unprocessed western blots. [file 41591_2022_1696_MOESM17_ESM.pdf]
